# The Impact of Patient Access to Electronic Health Records on Health Care Engagement: Systematic Review

**DOI:** 10.2196/56473

**Published:** 2024-11-20

**Authors:** Dalia Alomar, Maryam Almashmoum, Iliada Eleftheriou, Pauline Whelan, John Ainsworth

**Affiliations:** 1 Division of Informatics, Imaging and Data Sciences School of Health Sciences, Faculty of Biology, Medicine and Health, Manchester Academic Health Science System The University of Manchester Manchester United Kingdom; 2 Nuclear Medicine Department, Faisal Sultan Bin Eissa, Kuwait Cancer Control Center Kuwait City Kuwait

**Keywords:** electronic health records, personal health record, health care engagement, empowerment, patient experience, patient satisfaction, health care services, systematic review

## Abstract

**Background:**

Health information technologies, including electronic health records (EHRs), have revolutionized health care delivery. These technologies promise to enhance the efficiency and quality of care through improved patient health information management. Despite the transformative potential of EHRs, the extent to which patient access contributes to increased engagement with health care services within different clinical setting remains a distinct and underexplored facet.

**Objective:**

This systematic review aims to investigate the impact of patient access to EHRs on health care engagement. Specifically, we seek to determine whether providing patients with access to their EHRs contributes to improved engagement with health care services.

**Methods:**

A comprehensive systematic review search was conducted across various international databases, including Ovid MEDLINE, Embase, PsycINFO, and CINAHL, to identify relevant studies published from January 1, 2010, to November 15, 2023. The search on these databases was conducted using a combination of keywords and Medical Subject Heading terms related to *patient access to electronic health records*, *patient engagement*, and *health care services*. Studies were included if they assessed the impact of patient access to EHRs on health care engagement and provided evidence (quantitative or qualitative) for that. The guidelines of the PRISMA (Preferred Reporting Items for Systematic Reviews and Meta-Analyses) 2020 statement were followed for study selection, data extraction, and quality assessment. The included studies were assessed for quality using the Mixed Methods Appraisal Tool, and the results were reported using a narrative synthesis.

**Results:**

The initial search from the databases yielded 1737 studies, to which, after scanning their reference lists, we added 10 studies. Of these 1747 studies, 18 (1.03%) met the inclusion criteria for the final review. The synthesized evidence from these studies revealed a positive relationship between patient access to EHRs and health care engagement, addressing 6 categories of health care engagement dimensions and outcomes, including treatment adherence and self-management, patient involvement and empowerment, health care communication and relationship, patient satisfaction and health outcomes, use of health care resources, and usability concerns and barriers.

**Conclusions:**

The findings suggested a positive association between patient access to EHRs and health care engagement. The implications of these findings for health care providers, policy makers, and patients should be considered, highlighting the potential benefits and challenges associated with implementing and promoting patient access to EHRs. Further research directions have been proposed to deepen our understanding of this dynamic relationship.

## Introduction

### Background

Health information technologies (HITs) have played a pivotal role in reshaping the landscape of patient care in the 21st century, offering promises of enhanced efficiency and improved quality through advanced health information management practices [1]. Electronic health records (EHRs), a pivotal component of HITs, have revolutionized the management of patient information, promising to elevate the efficiency and quality of health care delivery through streamlined data access and use [2-4].

In addition, governments across the globe are championing the cause of patient-centered care, recognizing it as a fundamental tenet in the pursuit of health care excellence. Therefore, they are increasingly recognizing the pivotal role of EHRs in reshaping health care dynamics, highlighting their central role in the quest for patient empowerment and improved health care outcomes [5]. In 2015, a basic EHR system was adopted by 75% of US hospitals to enhance communication between patients and their health care providers (HCPs), reduce health care costs, and increase patients’ health care engagement [6]. The prioritization of citizen access to health records is a central focus of the MyHealthEData initiative by the US government. This is supported by initiatives by private entities such as Apple and Google, allowing patients to store their records on mobile devices. The 21st Century Cures Act enacted in the United States in December 2016 also includes crucial provisions that have the potential to substantially influence the availability, usability, and accessibility of health information for patients [7]. In the United Kingdom, primary care EHR providers, through TPP’s SystmOnline, Egton Medical Information Systems Patient Access, and In Practice Systems’ Patient Services, are actively providing patients with secure access to their health records. This access is further facilitated through the recently launched National Health Service app [5].

The significance of patient engagement in health care decision-making processes is now more pronounced than ever, with governments acknowledging its impact on both individual well-being and the overall quality of health care systems, enhancing patients’ right to be active participants in decision-making processes related to their health [8,9]. As nations strive to build health care systems that are responsive to individual needs, patient engagement, achieved by patients’ access to their EHRs, emerges as a linchpin, fostering a sense of ownership and partnership between patients and HCPs [10].

### The Evolving Role of EHRs

EHRs, also called electronic medical records (EMRs), are digital versions of patients’ comprehensive health information, including medical history, diagnoses, medications, treatment plans, immunization dates, allergies, radiology images, and laboratory test results. EHRs are designed to provide a centralized, electronic repository of health data that can be easily accessed and shared by authorized HCPs across different health care settings. The primary goal of EHRs is to improve the efficiency, quality, and safety of patient care regimen by facilitating accurate and timely access to pertinent health information, promoting care coordination among HCPs, and enhancing patient engagement in their health care journey [10].

Traditionally, EHRs were primarily viewed as tools for HCPs to record and retrieve patient information, with functionalities geared toward billing, documentation, and reimbursement purposes, thus streamlining the completion of various patient care tasks. The paradigm, however, has shifted as patients are now being granted direct access to their health records. This shift introduces a novel dynamic, empowering individuals to actively participate in their health care journey. Patient access to EHRs holds the promise of fostering a more collaborative and informed patient-HCP relationship, potentially influencing health care engagement in ways that extend beyond the traditional provider-centric model [11].

Patient access to EHRs empowers them with real-time and comprehensive health information, enabling informed decision-making aligned with personal preferences. This access facilitates seamless care continuity, offering a holistic view of one’s health care journey and contributing to personalized and coordinated care as well as adherence to appointments and treatment plans [12,13]. In addition, EHRs act as educational tools, enhancing health literacy and promoting proactive health management [14]. By fostering shared decision-making, patients become active contributors to their care, ensuring a patient-centered approach. Furthermore, EHR access strengthens patient advocacy, allowing individuals to actively engage with HCPs, seek second opinions, control their possible health risks, and contribute to discussions about their treatment options [15,16]. This redefined perspective on health care engagement involves providing patients access to EHRs, enhancing patient-centered care experiences, and fostering dynamic and collaborative relationships between patients and the health care system [17,18].

Despite the recognized benefits of EHRs and the increasing focus on patient-centered care, the specific impact of allowing patients access to their EHRs on improving their engagement with health care services remains a distinct and underexplored area. Therefore, this study aimed to systematically review the literature describing, examining, and evaluating the impact of patient access to EHRs on their health care engagement within different clinical settings (inpatient, outpatient, and emergency). Through this focused lens of patient-centered care, we aim to investigate the impact of patient access to EHRs on health care engagement, thereby contributing to the ongoing efforts to optimize health care quality and outcomes.

## Methods

### Research Question

The systematic review provided in this study was guided by the PRISMA (Preferred Reporting Items for Systematic Reviews and Meta-Analyses) 2020 statement [19]. Multimedia Appendix 1 shows PRISMA checklist used in this study. The research question was created based on the population, intervention, comparator, and outcome (PICO) framework [20], as shown in Textbox 1.

**Population, intervention, control, and outcome framework for the study strategy.**

**Population**

- The population involves individuals who have access to electronic health records (EHRs). It involves patients across various demographic groups and health care systems or with different medical conditions.

**Intervention**

- The intervention is patient access to EHRs.

**Comparator**

- The comparator involves comparing individuals with access to EHRs to those without such access.

**Outcome**

- The outcome is the improvement in engagement dimensions with health care services.

Accordingly, the research question of this review was defined as follows: Does patient access to EHRs improve engagement with health care services?

### Search Strategy and Sources of Information

The search strategy involved constructing specific terms related to “patient access,” “EHRs,” and “patient health care engagement” to ensure precision in the search results. The terms were expanded using Medical Subject Heading terms, synonyms, acronyms, abbreviations, and alternative spellings. These terms were combined using the Boolean operators AND and OR to optimize the search. Therefore, this phase involved identifying a comprehensive collection of studies relevant to the research question. This procedure encompassed *identification of the search keywords* and *definition of the search scope* [20].

During the *identification of the search keywords* phase, we established specific terms related to “patient access,” “EHRs,” and “patient health care engagement” to ensure precision in the search results. Following the approach recommended by Kitchenham et al [21], we broke down the research question into distinct units, treating each as a separate research unit. This involved encompassing Medical Subject Heading terms derived from the chosen databases, synonyms, acronyms, abbreviations, and alternative spellings, which were then amalgamated using the Boolean operators AND and OR to enhance the search strategy by locating the relevant studies. During the *definition of the search scope* phase, the source studies were acquired from the selected electronic databases (Ovid MEDLINE, Ovid Embase, PsycINFO, and CINAHL) through searches conducted up to November 15, 2023, using the formulated research keywords. The search strategies for the selected databases are presented in Multimedia Appendix 2.

### Eligibility Criteria

The eligibility criteria for inclusion in this study were carefully defined to ensure relevance and reliability in addressing the research question. Textbox 2 outlines the criteria for inclusion and exclusion in this study. Studies were included if they met the criteria, while those that did not meet the criteria were excluded.

**Eligibility criteria (inclusion and exclusion criteria).**

**Inclusion criteria**

- Article type: primary research studies including randomized controlled trials and observational studies (cohort and case control)
- Population: diverse patient populations across various demographics, medical conditions, and health care settings (inpatient, outpatient, and emergency)
- Intervention: examines the impact of patient access to electronic health records (EHRs) or electronic medical records (EMRs) or personal health record (PHR), as a main intervention, on health care engagement
- Focus: patient experiences and attitudes about the accessibility and use of EHRs (the demand side)
- Study design: quantitative, qualitative, and mixed methods designs
- Outcome measures: evaluates multiple dimensions of health care engagement (eg, involvement in health care decisions, adherence to treatment plans, communication, use of health care services, and experiences with patient-centered care)
- Publication type: peer-reviewed studies
- Publication date: studies published within the last 14 years (2010-2023)
- Language: studies published in English
- Availability: full text available or can be retrieved

**Exclusion criteria**

- Article type: secondary research (eg, reviews and meta-analyses) or other nonprimary research types (eg, editorials)
- Population: studies that lack suitable comparators or control groups essential for evaluating the impact of patient access to EHRs on health care engagement
- Intervention: studies that do not address patient access to EHRs, EMRs or PHR as a main intervention
- Focus: practitioner experiences and attitudes about EHR use (the supply side)
- Study design: studies that do not use quantitative, qualitative, or mixed methods designs
- Outcome measures: studies that do not evaluate the relevant dimensions of health care engagement as the main outcome
- Publication type: non–peer-reviewed publications (eg, editorials, book chapters, opinion pieces, commentaries, and conference abstracts)
- Publication date: studies published outside the 2010-2023 time frame
- Language: studies published in languages other than English
- Availability: studies with unavailable full text or those that cannot be retrieved

### Selection Process

All studies identified through the database searches were exported to web-based EndNote (Clarivate Analytics) for screening and removing any duplicates. The selection process followed a systematic approach to identify and include relevant studies. It was conducted in accordance with the PRISMA 2020 statement to ensure transparency and reproducibility. First, a comprehensive search was conducted using 4 selected electronic databases, including MEDLINE, CINAHL, PsycINFO, and Embase. The search used the predefined keywords and eligibility criteria. Then, duplicates were eliminated, which arise due to the presence of certain studies in multiple databases. Titles and abstracts of the remaining studies were screened independently by 2 authors (DA and MA) to assess their relevance to the research question and eligibility criteria. Accordingly, all studies that did not meet these criteria were excluded, while studies passing this initial screening underwent a thorough full-text review by the 2 authors. These 2 authors assessed the entire text of the articles to confirm their eligibility and relevance. Nonrelevant studies were removed. Any discrepancies in study selection were resolved through discussion and consensus among the review team members.

### Data Collection Process

Following the final selection of eligible studies by the 2 authors, data were systematically extracted by the primary author (DA). The extracted data encompassed various aspects, including study characteristics (eg, author, publication year, country, aim, intervention specifics, study design, study setting, patient demographics, and sample size), study outcome measures, key findings pertaining to health care engagement and the systematic review’s research question, and quality of the study (based on the evidence strength). The accuracy of the extracted data was verified by the research team.

### Risk of Bias and Quality Assessment of the Included Studies

After the final selection of the included studies, the risk of bias was assessed using the Mixed Methods Appraisal Tool (MMAT) [22]. It is used for assessment in reviews encompassing quantitative (randomized controlled trial [RCT], nonrandomized, or descriptive), qualitative, and mixed methods studies. The MMAT advises against assigning a singular score based on the evaluation [22]. Following a previous study [23], we used the κ statistic to evaluate the quality of each study, supporting our final decisions based on the predetermined inclusion and exclusion criteria. On the basis of the fulfillment of criteria, the studies were categorized as high, medium, or low quality. A study attained a high-quality classification if it met all 5 MMAT criteria, a medium quality if 3 or 4 criteria were met (ie, meeting some criteria), and a low-quality classification if only 1 or 2 criteria were met (ie, meeting minimum criteria) [23].

### Data Synthesis

Data extracted from the included studies were synthesized and analyzed using a narrative synthesis. A narrative synthesis is fitting and applicable in this study because it incorporates qualitative, quantitative, and mixed methods findings [24]. Studies with comparable outcomes, concerning dimensions of patient health care engagement, were pooled for summarization. The findings were presented by categorizing the results of different studies based on common outcomes of the patient health care engagement. This involved determining the number and percentage of studies associated with each outcome and leveraging these findings to draw conclusions. Given the variability observed in EHRs, health care settings, and patient populations, a meta-analysis was deemed inappropriate. Data analysis was conducted by one author (DA), and any discrepancies were resolved through discussions with the other author (MA). Through the analysis, we identified categories within patient health care engagement, as shown in Table 1.

**Table 1:** Main categories and dimensions of patient health care engagement outcomes (N=18).

Categories and dimensions		Studies, n (%)	Reference
**Treatment adherence and self-management**			
	Adherence to prescribed treatments and medications plans	7 (39)	Zarcadoolas et al [25] Wang et al [26] Nazi et al [27] Suija et al [28] Wass et al [29] Spratt et al [30] van der Vaart et al [31]
	Self-monitoring or tracking disease and health parameters over time	4 (22)	Klein et al [32] Ryu et al [33] Haggstrom et al [34] Hanna et al [35]
	Self-management of diseases by using self-management tools (eg, mobile apps or PHRs^a^)	2 (11)	Fuller et al [36] Hanna et al [35]
**Patient involvement and empowerment**			
	Improved accessibility to or usability of health care information	6 (34)	Nazi et al [27] Suija et al [28] van der Vaart et al [31] Haggstrom et al [34] Hanna et al [35] Moll et al [37]
	More involvement in treatment and active participation in disease and medication management decisions (eg, patient-generated data and refilling prescriptions)	4 (22)	Zarcadoolas et al [25] Suija et al [28] Wass et al [29] van der Vaart et al [31]
	Enhanced patient agency and responsibility for their care	2 (11)	Wass et al [29] Hanna et al [35]
	Confidence in managing one’s health	1 (5)	Wolff et al [38]
	More informed (health literacy) and better understanding of their condition (disease and treatment)	8 (44)	Klein et al [32] Nazi et al [27] Suija et al [28] Wass et al [29] van der Vaart et al [31] Wolff et al [38] Moll et al [37] Mák et al [39]
**Health care communication and relationship**			
	Better prepared for office visits	1 (5)	Wolff et al [38]
	Improved frequency and quality of communication between patients and HCPs^b^ regarding disease monitoring	10 (56)	Klein et al [32] Zarcadoolas et al [25] Nazi et al [27] van der Vaart et al [31] Fuller et al [36] Wolff et al [38] Hanna et al [35] Moll et al [37] Wagner et al [40] Ibrahim et al [41]
	Discussion and collaboration between patients and HCPs about patient treatment plans	4 (22)	Klein et al [32] Nazi et al [27] Spratt et al [30] Wolff et al [38]
	Trust in HCPs and improved patient-HCP relationship	4 (22)	Klein et al [32] Nazi et al [27] van der Vaart et al [31] Fuller et al [36]
	Shared decision-making	4 (22)	Wang et al [26] Ryu et al [33] Haggstrom et al [34] Hanna et al [35]
**Patient satisfaction and health outcomes**			
	Perceived effectiveness of treatments	4 (22)	Klein et al [32] Wang et al [26] Ryu et al [33] Wagner et al [40]
	Improvement in health conditions and quality of health care and life	4 (22)	Wang et al [26] Nazi et al [27] van der Vaart et al [31] Hanna et al [35]
	Functions tailored to personalized preferences	3 (17)	Zarcadoolas et al [25] Krist et al [42] Haggstrom et al [34]
	Achievement of health-related goals (eg, health risk assessment)	2 (11)	Krist et al [42] Haggstrom et al [34]
	Overall satisfaction with health care services	2 (11)	Mák et al [39] Ibrahim et al [41]
**Use of health care resources**			
	Frequency of health care visits	1 (5)	Zarcadoolas et al [25]
	Efficient use of health care and information resources (review, correct, and update health information)	1 (5)	Krist et al [42]
	Use of preventive care services	1 (5)	Krist et al [42]
**Usability** **concerns and barriers**			
	Privacy and security concerns	2 (11)	Zarcadoolas et al [25] Haggstrom et al [34]
	Technical barriers in accessing or using health records features	3 (17)	Zarcadoolas et al [25] Suija et al [28] Spratt et al [30]
	Barriers in comprehension of health information or medical terms	3 (17)	Zarcadoolas et al [25] Wass et al [29] Suija et al [28]
	Medications and follow-up concerns after discharge from the hospital	1 (5)	Fuller et al [36]

^a^PHR: personal health record.

^b^HCP: health care provider.

## Results

### Study Selection

**Figure 1:**
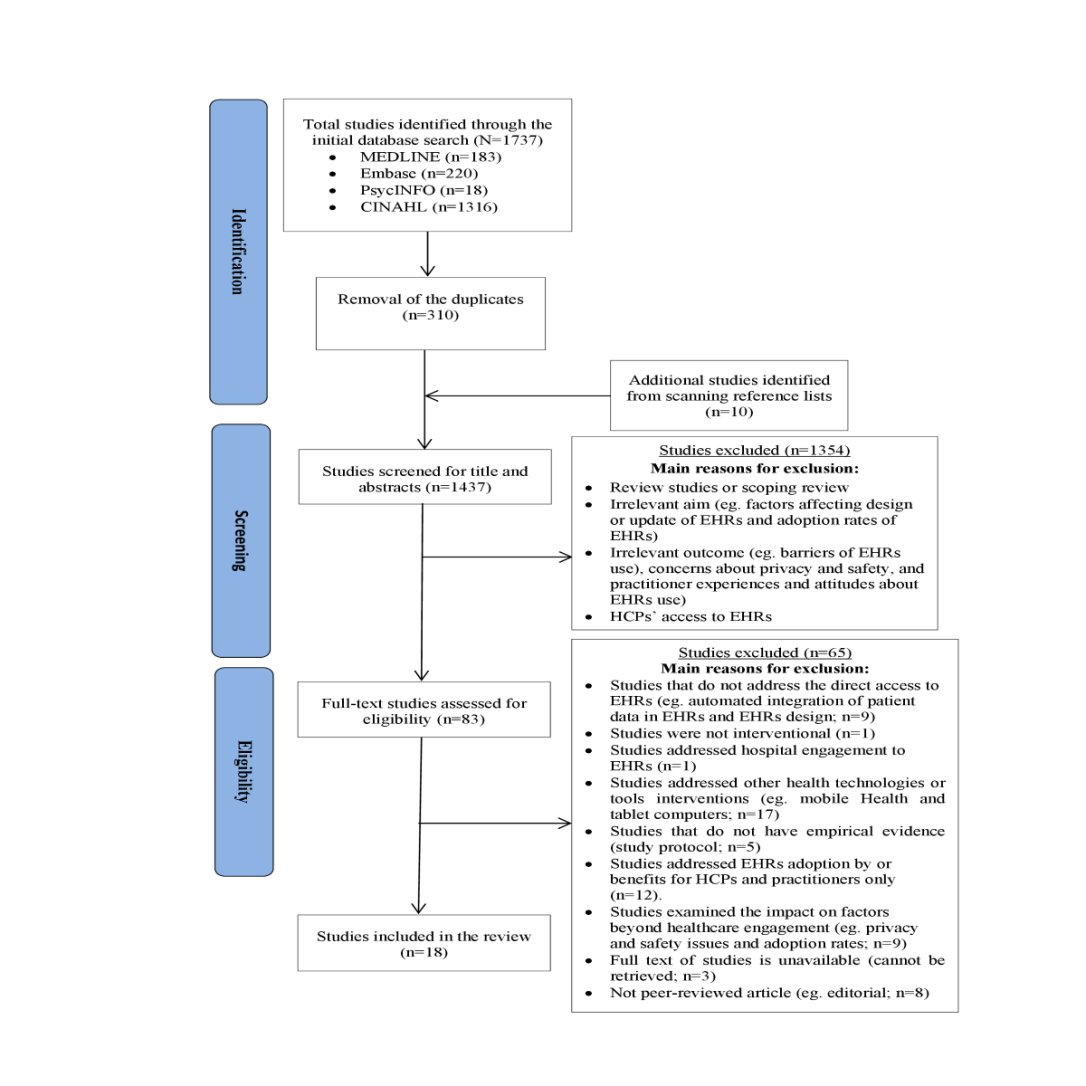
PRISMA (Preferred Reporting Items for Systematic Reviews and Meta-Analyses) 2020 statement flowchart for the search and selection process of the included studies. EHR: electronic health record; HCP: health care provider.

The electronic search from the 4 selected databases retrieved a total of 1737 study records, of which 183 (10.54%) were from Ovid MEDLINE, 220 (12.66%) were from Ovid Embase, 18 (1.04%) were from Ovid PsycINFO, and 1316 (75.76%) were from EBSCOhost CINAHL. Following the removal of duplicate records (n=310, 17.74%) and addition of 10 articles based on scanning reference lists of the articles initial searched, 1437 (82.26%) studies remained that underwent assessment during title and abstract screening. Of these 1437 studies, 83 (5.77%) met the eligibility criteria for full-text screening, and the final number of studies included in the review was 18 (1.25%). The selection process and rationale for choosing these studies are shown in Figure 1.

### Characteristics of the Included Studies

Multimedia Appendix 3 [25-42] provides a summary of the characteristics of the studies that have been included. All included studied aimed at investigating or describing the impact of patient access to or the implementation of EHRs with their different applications and platforms on patient engagement, including their attitudes, perceptions, experience, empowerment, and satisfaction. The 18 studies included in the review spanned various global regions: 8 (49%) were from the United States, 2 (11%) were from Sweden, 1 (5%) was from Germany, 1 (5%) was from China, 1 (5%) was from Estonia, 1 (5%) was from South Korea, 1 (5%) was from the Netherlands, 1 (5%) was from Australia, 1 (5%) was from Canada, and 1 (5%) was from Malaysia.

Of the 18 studies, qualitative methods (focus groups, interviews, semistructured interviews, and observations) were used in 3 (17%) of studies; quantitative methods (surveys, questionnaires, laboratory test, and mobile phone app recording activity log [eg, MyHealthKeeper]) were used in 10 (56%) of studies; while 5 (27%) studies adopted a mixed approach. Regarding the study designs, the studies used a RCT (4/18, 22%), cross-sectional (2/18, 11%), and pretest-posttest implementation design (2/18, 11%), and the remaining studies used observational, case report, or exploratory study with a retrospective cohort design.

For participants demographics, the studies varied regarding their participants; few studies were focused on both patients and physicians (eg, dermatologists), while most targeted patients only. All included studies shared that the patient sample encompassed adult patients (aged 21-83 years), with sex diversity (male individuals and female individuals), had experience with computers and use the internet, diagnosed in most studies with a specific disease (eg, chronic obstructive pulmonary disease [COPD], diabetes, uncontrolled hypertension, or metabolic syndrome), and they enrolled in or accessed EHRs with various applications and web-based portals or platforms during the study period. The clinical setting of the included studies encompassed various settings, including inpatient, outpatient, and ambulatory clinics.

Within the scope of patient access to EHRs, the 18 included studies revealed a spectrum of interventions related to patient access to or implementation of EHRs either via patient portals (n=5, 28%), a specific mobile app such as MyChart or MyHealthKeeper (n=2, 11%), or other web-based platforms (n=11, 61%). Notably, a subset of studies concentrated on disease-specific applications, such as using EHRs in psoriasis (1/18, 5%) or diabetes care (1/18, 5%). Studies describing patients accessing their medical records either through personal health records (PHRs) tethered to EHRs or EMRs (5/18, 28%) or interactive PHRs (IPHRs; 1/18, 5%) stood out as approaches generating tailored recommendations from EHRs or EMRs, emphasizing personalized health care. Other studies explored unique interventions, such as web-based coaching programs (1/18, 5%), the MyHealtheVet (MHV) Pilot Program (1/18, 5%), and the implementation of a discharge video checklist (1/18, 5%), all aiming to enhance patient engagement through electronic records.

All included studies [25-42] addressed evaluating ≥1 dimensions and measures of health care engagement outcomes because of patient EHRs access, including patient involvement in health care decisions, adherence to treatment plans, communication with HCPs, use of health care services, and experiences with patient-centered care. The identified categories of theses dimensions of health care engagement outcomes are shown in Table 1.

Multimedia Appendix 4 shows the quality of the included studies [25-42]. Just 1 (5%) of the 18 studies was considered as having low quality [28]. Despite its methodological limitations, such as a small sample size and issues with data quality inspection, the study’s findings offer qualitative depth that complements quantitative evidence in understanding the complexities of health data use via access to the EHRs. Therefore, this paper needs to be documented in this study. Specifically, Suija et al [28] identified 3 crucial themes for the experiences of patients and primary care physicians with health data, particularly during the COVID-19 pandemic. These themes included access to health records, experiences with using data in health records, and the use of patient-generated data, providing unique insights into the perspectives of both patients and physicians.

### Quality (Risk of Bias) of the Included Studies

Multimedia Appendix 4 [25-42] summarizes the outcomes of the quality assessment conducted for the included studies. Of the 18 studies, 14 (78%) were classified as high quality, meeting all 5 MMAT criteria: 1 (5%) qualitative, 8 (45%) quantitative, and 5 (28%) mixed methods studies. In addition, 3 (17%) of the 18 studies were deemed medium quality, fulfilling 3 or 4 of the MMAT criteria: 1 (5%) qualitative and 2 (12%) mixed methods studies. Of the 18 studies, 1 (5%) with a qualitative approach was identified as low quality, meeting only 2 of the MMAT criteria. Given the exploratory nature of this review and its limited research evidence and in accordance with the suggestion of Hong et al [22], we opted not to exclude the study meeting only 2 of the MMAT criteria from the final review.

### Synthesis of the Results

#### Overview

Table 1 shows how patient engagement to health care services is improved through their access to EHRs. We categorized the identified dimensions and outcomes of patient engagement that were affected by patient EHRs access by grouping them according to their shared characteristics, as outlined in prior studies [1-24]. These dimensions were classified into 6 categories based on the nature of engagement: treatment adherence and self-management, patient involvement and empowerment, health care communication and relationship, patient satisfaction and health outcomes, use of health care resources, and usability concerns and barriers.

#### Categories of the Dimensions of Patient Health Care Engagement Outcomes Affected by EHRs Access

As mentioned in the *Synthesis of the Results* section, the identified dimensions and outcomes of patient engagement that were affected by patient access to EHRs were classified into 6 main categories, as shown in Table 1. The following sections synthesize the categories of those dimensions.

#### Treatment Adherence and Self-Management

As shown in Table 1, there are 3 identified dimensions of patient engagement in this category. The most cited dimension of them is the adherence to prescribed treatments and medication plans. It is cited in 7 (39%) of the 18 studies [25-31]. Adherence to prescribed treatments and medication plans refers to the extent to which patients follow the recommendations and instructions provided by HCPs regarding their medical treatments, including medication regimens. Adherence is a critical factor in achieving positive health outcomes. It directly influences the effectiveness of treatments, disease management, and overall well-being. Poor adherence can lead to treatment failure, worsening of health conditions, and increased health care costs.

Patient access to EHRs plays a pivotal role in influencing adherence to prescribed treatments. With access to their health information, including medication lists, dosage instructions, and treatment plans, patients found that they could gain a clearer understanding of their prescribed regimens [28,29,31]. This increased awareness empowers patients to take an active role in managing their health. Patient access to their medical records through a PHR connected to an EHR, which included wellness reminders, helped them take action and adhere to their treatment plans [27]. In addition, EHRs can facilitate automated reminders for medication schedules and upcoming appointments, annual visits, and screenings. These reminders serve as prompts for patients to adhere to their prescribed plans consistently [25]. For evaluation of a web-based coaching program using EHRs for patients with COPD, Wang et al [26] illustrated that this intervention comprises web-based EHRs containing extensive information about preventive measures, treatment options, pulmonary rehabilitation, and variations in the disease. These resources motivated patients to actively participate in healthy behaviors and enhance adherence to prescribed medications, oxygen therapy, and respiratory exercises. Moreover, The Duke PillBox application, a medication management application integrated into the EHRs’ patient portal using Substitutable Medical Applications and Reusable Technologies on Fast Healthcare Interoperability Resources technology, is considered beneficial due to enhanced patient interactions, leading to improved adherence as patients follow prescribed medication regimens [30].

In total, 4 (22%) of the 18 studies illustrated that patient access to their EHRs significantly helped in disease and health parameters monitoring and tracking over time [32-35]. Using an EHR enabled patients to monitor their disease progression over time [32]. Personally controlled EHR (PCEHR) enabled patient to review and self-monitor their health status and treatment [35]. MyHealthKeeper, an EHR-tethered PHR application, demonstrated the effectiveness of a health tracker system managed by patients and guided by clinicians [33]. The MHV program, integrated into PHRs, also empowered users to monitor their health by incorporating various types of self-reported health information, including vital signs, laboratory results, tests, and records of food and activity journals [34]. Regarding the active engagement of individuals in managing and controlling aspects of their health related to a specific medical condition, it is reported in 2 (11%) of the 18 studies that patient access to EHRs plays a significant role in diseases self-management by using self-management tools (eg, mobile apps or PHRs) [35,36].

#### Patient Involvement and Empowerment

Patient involvement and empowerment refer to the active participation, engagement, and empowerment of individuals in their own health care processes and decision-making. Empowerment involves providing individuals with the knowledge, skills, and resources necessary to actively participate in discussions about their health, set personal health goals, and contribute to the decision-making process. Five dimensions of patient engagement are identified in studies for this category.

Of the 18 included studies, 6 (34%) addressed the improved accessibility to health care information using EHRs. PHRs enhanced the value of users’ data by ensuring information is more accessible and understandable [34]. Using the MHV program integrated into PHRs, patients engaged with self-instructional materials. This facilitated their ability to find pertinent information through the health education library; input data using the website’s Healthelog features; search for information on specific diseases, conditions, or treatments; and engage in discussions with their HCPs. The features most valued by users included accessing portions of their medical record, reviewing prescription history, and checking upcoming appointments [27]. Suija et al [28] demonstrated that both patients and physicians recognized the importance of accessing health records. Patients highlighted the significance of accessing documents such as hospital discharge summaries, consultation responses, and test results. In contrast, physicians emphasized the need for convenient access to past patient health records to effectively prepare comprehensive treatment plans. The hospital-based patient web portal, offering remote access to EMRs, also demonstrated ease of use and high utility, with minimal reported issues [31]. The PCEHR provided patients with direct and immediate access to their health information without the need for intermediaries, such as HCP gatekeepers. This empowered patients to use the information collaboratively with HCPs in making informed choices about their health care [35]. Moreover, Moll et al [37] showed that patient access to EHRs through Journalen, the Swedish national patient portal, facilitated retrieving their test results, immunization history, and visit details, improving their accessibility and usability of their health care information.

There were 4 (22%) out of 18 studies that focused on patient engagement through more involvement in their treatment and active participation in disease and medication management decisions [25,28,29,31]. Patient portals played a crucial role in promoting patient empowerment, particularly in medication management by using features such as making appointments, refilling prescriptions, and contributing patient-generated data [25]. Suija et al [28], using individual semistructured interviews with patients and physicians, reported that patients expressed a willingness to generate crucial data necessary for medical decisions, such as completing questionnaires before appointments to systematize their complaints and save time during consultations. Physicians also acknowledged the value of patient-generated data and considered it valuable when making treatment decisions. The accessibility of EHRs is linked to heightened patient involvement in their treatment. This increased accessibility results in patients being more likely to follow the advice provided by HCPs, have better recall of information, understand discussions during appointments more clearly, and communicate more effectively with HCPs about their situation. As a result, they can actively participate in managing their diseases and medication decisions [29,31].

There were 8 (44%) out of 18 studies that highlighted that patient access to EHRs contributes to increased patient awareness and understanding of their condition, fostering improved health literacy [27-29,31,32,37-39]. Patient access to EHRs enhances comprehension of their medical problems and treatments [27]. This improved understanding extends to a better grasp of their overall care, fostering feelings of being more informed and secure [37,38]. The access to EHRs facilitated the understanding, discussion, and coordination of documented information, making it easier for patients to comprehend what was said during appointments and communicate with HCPs about their situation (diseases and instructions for self-care) [28,29]. Patients also found it intriguing to compare their data with those of others, enabling more informed discussions with HCPs [32]. Among patients who logged into the hospital-based patient web portal with home access to EMR, 37% reported having increased knowledge about their diseases and treatment [31]. However, in exploring the impact of web-based access to laboratory test results in British Columbia on patient experiences, Mák et al [39] conducted an exploratory study with a retrospective cohort design, revealing that while both service users and the comparison group reported understanding their test results, the rate was slightly lower for service users (75.55% vs 84.69%).

Patient access to EHRs significantly enhanced patient agency and responsibility for their care [29,35]. Web-based access to EHRs made it easier for patients to take greater responsibility for their care [29]. Use of Medenote as a PCEHR promoted patient agency by enhancing their capacity for self-management and making independent, informed choices about their health [35]. Furthermore, according to Wolff et al [38], patients exhibited heightened confidence in managing their health.

#### Health Care Communication and Relationship

Patient access to EHRs plays a significant role in improving patient engagement through enhancing the physician-patient communication and relationship. There are 5 identified dimensions for this patient engagement outcome. Using EHRs, patients felt better prepared for office visits [38]. Furthermore, more than half of the studies (10/18, 56%) focused on the crucial effect of patient access to EHRs on the frequency and quality of communication between patients and HCPs regarding disease monitoring [25,27,31,32,35-38,40,41]. This access played a crucial role in enhancing their communication with HCPs [25,27,31,35,37]. The frequency of this communication is associated with the regular use of PHRs or EHRs [40,41]. It is positively impacted by patient access to visualized data [32]. Patients perceive that when preparing for discharge from the hospital, the integration of digital health tools with EHRs such as discharge videos, checklists, and postdischarge SMS text messaging fostered communication with their health care team [36]. In addition, the electronic delivery of doctors’ visit notes to patients and authorized care partners through OpenNotes (Deaconess Medical Center) contributes to enhanced communication with HCPs [38].

Several studies (4/18, 22%) found that access to EHRs not only enhances communication and relationships between HCPs and patients but also facilitates collaborative discussions about patient treatment plans, particularly when based on visualized data [32]. The discussion of medication reconciliation, including indications, side effects, and barriers to use, becomes vital for improved communication when patients have access to EHRs [27,30]. Wolff et al [38] illustrated that patients and care partners reported better agreement about patient treatment plans and more productive discussions regarding patient care, emphasizing the positive impact of EHR access on collaborative health care discussions.

In total, 22% (4/18) of the studies revealed that shared decision-making is a crucial aspect of HCP-patient communication, which is influenced by patient access to EHRs [26,33-35]. It is reported that the use of a web-based coaching program using EHRs is instrumental in fostering the sharing of patients’ medical information among hospital and community nurses, facilitating dynamic management, and enabling follow-up analysis of patients’ diseases [26]. MyHealthKeeper, an EHR-tethered PHR application, allows the seamless sharing of life log data between clinicians and patients [33]. Patient access to the MHV program integrated into PHRs revealed preference for information sharing with HCPs among most users [34]. In addition, through access to PCEHR, patients reported their ability to use health information in conjunction with HCPs, empowering them to make informed choices about their health care [35].

In total, 17% (3/18) of the studies found that trust in HCPs and improved patient-provider relationships are crucial dimensions influenced by patient access to EHRs [31,32,36]. It is the result of the improved communication between HCPs and patient. Patient access to EHRs and discussions centered on visualized data enhanced communication and, consequently, improved the relationship between patients and physicians [32]. Furthermore, the trust of patients in HCPs was heightened through patient access to EMRs [31,36].

#### Patient Satisfaction and Health Outcomes

This category of patient health care engagement encompasses the overall contentment of patients with their health care experiences and the impact of these experiences on their well-being. It examines the extent to which patients are pleased with the health care services they receive and explores the resulting effects on their health status.

There were 4 (22%) out of 18 studies that concluded that patient access to EHRs affects their satisfaction through perceived effectiveness of treatments [26,32,33,40]. Patients reported that using an EHR resulted in an enhancement in the quality of their treatment [32]. When Wang et al [26] investigated the effectiveness of a web-based coaching program using EHRs for improving physical function in patients with COPD across 2 hospitals in China, they indicated that this program could delay the decline in lung function, reduce dyspnea, and enhance physical capacity. Through analyzing data on health outcome measures such as weight change, changes in blood biochemical parameters (cholesterol, triglycerides, high-density lipoprotein cholesterol, and low-density lipoprotein cholesterol), Ryu et al [33] revealed that MyHealthKeeper PHR application was found to improve patient clinical profiles, as evidenced by weight loss and lower triglyceride levels [33]. In contrast, Wagner et al [40] conducted an intervention involving a PHR tethered to the patient’s EMR for 453 patients, including those with metabolic syndrome, in ambulatory clinics. Their results showed no observed impact of the PHR on blood pressure. However, in the subanalysis of that study, a further examination of patients participating in the intervention, specifically those who identified themselves as active users of the PHR, revealed a decrease of 5.25 points in diastolic blood pressure.

Improvement in health conditions and quality of health care and life was reported in 4 (22%) of the 18 studies [26,27,31,35]. Patient access to EHRs has improved the quality of care and life through various applications and platforms, including a web-based coaching program using EHRs [26], a web portal [31], PCEHRs [35], and MHV (a PHR connected to EHRs). These resources, which include wellness reminders, help patients take action, adhere to their treatment plans, and enhance communication with their HCPs [27].

Functions tailored to personalized preferences are a significant aspect of patient satisfaction influenced by access to EHRs, as reported in 17% (3/18) of the studies [25,34,42]. Accessing EHRs through patient portals positively influenced health outcomes by providing personalized reminders for scheduled appointments, annual visits, and screenings [25]. The IPHR demonstrated its capability to offer personalized recommendations for patients [42]. Usability tests conducted on PHRs provided valuable insights into tailoring functions according to individual preferences [34].

Achievement of health-related goals is a valuable dimension in patient satisfaction that is affected by patient access to EHRs, as reported in 2 (11%) of the 18 studies [34,42]. It concerns about enhancing various aspects of patient daily life, such as physical, mental, and social well-being. Using EMRs, the IPHR effectively involved patients in preventive care by conducting a concise health risk assessment. This assessment collects information that may not be well documented electronically, including health behaviors and psychosocial measures [42]. The MHV program, integrated into PHRs, also enabled patients adapting to posttraumatic stress disorder and healthy eating [34].

In 11% (2/18) of the studies, patient access to EHRs was found to improve the overall satisfaction with health care services [39,41], especially a larger proportion of users of the web-based access to laboratory test results service received their results more promptly, typically within “a few days,” in contrast to just over a third of the comparison group. This discrepancy in result delivery times contributed to higher overall satisfaction levels among service users [39].

#### Use of Health Care Resources

Use of health care resources is considered an important category of patient engagement impacted by patient access to EHRs. It involved the frequency of health care visits via EHRs. According to 6% (1/18) of the studies, accessing EHRs through patient portals resulted in longer doctor’s visits, allowing patients more time with their doctors [25]. This contributed to improving the effectiveness of visits with HCPs.

Moreover, this access to EHRs is found to achieve efficient use of health care and information resources in 5% (1/18) of the studies [42]. Krist et al [42] revealed that using EMRs, the IPHR effectively established an open database connection to link with the EMR of the patient’s designated clinician, extracting pertinent clinical data. This enables patients to access information such as their medical history, medications, immunizations, and test dates. The platform empowers patients to review, correct, and update their information as needed.

Another dimension of efficient use of health care resources is the use of preventive care services. Krist et al [42] revealed that by using EMRs, the IPHR effectively involved patients in preventive care through enabling patient access to results related to preventive care, such as recommendations for chronic care and managing high cholesterol, achieving a use rate that varied from 1.5% to 28.3% across different health care practices.

#### Usability Concerns and Barriers

Some of the included studies reported that there were some usability concerns and barriers resulting from using or accessing EHRs. Privacy and security concerns were raised by 11% (2/18) of studies [25,42]. It is found that PHR registration should strike a balance between simplicity and security [34]. Concerns related to the privacy and security of web-based records, such as potential damage or hacking, were expressed by a small number of participants using EHRs via patient portals [25].

Technical barriers in accessing or using health records features are reported in 17% (3/18) of the studies [25,28,30]. Consumers expressed apprehensions about various obstacles to use, including intricate visual layouts and suboptimal usability features [25]. Patient access to documents in health records through the patient portal is deemed intricate [28]. Usability challenges encompass EHR integration, concerns with the EHR-portal communication interface, as well as issues related to browser access and compatibility, affecting both patients and HCPs [30].

Furthermore, barriers related to comprehension of health information or medical terms are reported in 17% (3/18) of the studies [25,28,29]. Consumers expressed concerns about the complexity of language used in EHRs [25]. Challenges such as patients grappling with medical terminology and the necessity for rigorous inspection of medical documents were noted [28]. In a study by Wass et al [29], only 9% of participants reported being worried or upset about the information in the EHRs, with some finding it challenging to comprehend due to the inclusion of medical terms. In addition, concerns regarding medications and follow-up after discharge from the hospital were raised in 5% (1/18) of the studies [36].

## Discussion

### Principal Findings

This systematic review comprises 18 studies exploring the impact of patient access to EHRs on various aspects of health care engagement. Our findings revealed that most studies reported positive findings for patient health care engagement, including enhanced patient empowerment and involvement, improved adherence to treatment plans and self-management, increased patient confidence in managing their health, promoted health care communication and relationship and trust between HCPs and their patients, effective use of health care resources, and improved patient satisfaction and health outcomes. In addition, some studies raised some usability concerns and barriers for EHRs access.

This comprehensive analysis directly addresses our research question, enhancing our understanding of the role EHRs play in shaping patient engagement and guiding future research and health care practices toward more patient-centered care. However, it is crucial to acknowledge the diversity among the studies, encompassing variations in interventions related to EHRs access, types of applications and platforms used for EHRs access, health care settings, methodologies, and outcome measures. This diversity adds complexity to the interpretation of findings and underscores the need for a nuanced understanding of the implications of patient access to EHRs.

A broad range of interventions, all related to EHRs access, included access to EHRs via patient portals, web-based coaching programs using EHRs, use of PHRs tethered to EHRs, integration of digital health tools with EHRs (eg, discharge videos, checklists, and SMS text messaging), use of mobile apps (eg, Duke PillBox, MyHealthKeeper, and MHV) for accessing EHRs, implementation of health tracker systems (eg, Misfit), open access to laboratory test results through EHRs, and delivery of physician’s visit notes electronically via OpenNotes. Similarly, the included studies encompassed several types of EHRs, including EMRs accessed through a web portal, web-based access to health information through PHRs (eg, via MHV program integrated into PHRs), PCEHRs, Duke PillBox application (integrated into EHRs patient portal), Journalen (personal accessible EHRs), and IPHR. This variety in EHR-related interventions and EHR types collectively represents a diverse array of strategies and platforms aimed at enhancing patient engagement and health care outcomes as well as emphasizes the multifaceted nature of patient engagement in the context of electronic health information systems.

### Comparison With the Literature

Our findings in this systematic review indicated that patient access to EHRs has a significant influence on diverse categories and dimensions of patient health care engagement. The six main categories or themes that emerged from the included studies encompassed the following: (1) treatment adherence and self-management, (2) patient involvement and empowerment, (3) health care communication and relationship, (4) patient satisfaction and health outcomes, (5) use of health care resources, and (6) usability concerns and barriers. Our findings are consistent with the Patient Portal Engagement Framework developed by Zhou et al [43], which includes 4 levels of engagement: Inform Patients, Involve Patients, Partner with Patients, and Support Ecology of Care.

However, the 6 categories or themes identified in our review extend beyond the Patient Portal Engagement Framework by providing a more granular view of patient engagement outcomes. This comprehensive set of outcome measures for patient health care engagement, as highlighted in Table 1, offers a broader perspective than what is typically covered in the literature. While some studies may focus on 1 or 2 dimensions of patient engagement, our work integrates multiple dimensions, thereby providing a holistic view of patient engagement outcomes.

Moreover, the 6 categories or themes have not been previously described in the literature as a unified framework, making this a novel contribution of our systematic review. This framework provides a structured approach to understanding the multifaceted nature of patient engagement resulting from EHRs accessibility, encompassing both benefits and challenges. This unique categorization can serve as a valuable contribution for knowledge and practical applications in enhancing patient engagement through EHRs. This comprehensive framework can be discussed as follows.

Patient access to EHRs positively affects treatment adherence and self-management for patients. This access empowers patients by providing information on medications, dosage instructions, and treatment plans [28,29,31]. This heightened awareness leads to active patient participation in health care management [27]. EHRs, especially when integrated into patient portals or PHRs, prove beneficial through wellness reminders, encouraging patients to adhere to treatment plans consistently [25,30].

Our findings confirm those of previous studies, which consistently show that EHRs are powerful tools for self-monitoring and self-management of disease and health parameters over time. Patients using EHRs can longitudinally track disease progression [32]. The PCEHR system allows patients to review and self-monitor their health status and treatment [35]. MyHealthKeeper and MHV also empower users to monitor their health through self-reported information, enhancing engagement [33,34]. In self-management of diseases through tools such as mobile apps or PHRs, patient access to EHRs plays a significant role [35,36], especially for patients with chronic disease (eg, patients with diabetes) [44]. Patients actively engage in managing their health, supported by the functionalities provided by EHRs [35,36]. This is in line with prior studies that indicated that EHRs, when integrated into a patient-centered self-management strategy, have the potential to empower patients for more effective self-management of their diseases and can help address challenges that contribute to unsuccessful treatment outcomes [45]. For example, individuals using the Canadian MyChart PHRs began associating their dietary choices with their laboratory test results, specifically sodium levels [46].

The studies consistently demonstrated that patient access to EHRs enhances the accessibility and usability of health care information. PHRs, such as MHV, were instrumental in providing patients with self-instructional materials, contributing to a better understanding of their health [28]. The hospital-based patient web portal and PCEHR further extended the ease of access to health records, promoting patient engagement [31,35]. Therefore, these findings align with the broader theme of empowering patients through improved access to their health care information.

Patient portals emerged as facilitators of active patient involvement, especially in medication management. Patients expressed a willingness to generate crucial personalized health data, emphasizing the importance of their active participation in medical decisions [25,28,29,31]. The increased accessibility to EHRs was linked to heightened patient involvement, indicating that informed patients are more likely to actively engage in decisions related to their treatment plans.

The studies consistently highlighted that patient access to EHRs contributes to enhanced patient agency and a greater sense of responsibility for their care. Web-based access to EHRs made it easier for patients to take control of their health [29]. For instance, Medenote, as a PCEHR, played a pivotal role in promoting patient agency, allowing them to make independent and informed choices about their health [35]. Therefore, this finding underscores the transformative impact of EHR access on patients’ roles in managing their health care. Wolff et al [38] revealed a crucial aspect of patient empowerment—heightened confidence in managing their health. This aligns with the broader narrative that patient access to EHRs not only provides information but also instills a sense of confidence in patients about managing their health effectively.

The studies consistently demonstrated that patient access to EHRs contributes to increased patient awareness and understanding of their condition. Patients found it intriguing to compare their data with others, fostering more informed discussions with HCPs [32]. However, it is noteworthy that the exploratory study by Mák et al [39] revealed slightly lower rates of understanding test results among service users, indicating potential variations in health literacy outcomes.

Regarding the impact of EHRs access on health care communication and relationship, the findings revealed multifaceted impact. Patients expressed feeling better prepared for office visits when granted access to EHRs. This preparation likely stems from their ability to review and familiarize themselves with their medical records, enabling more informed and productive discussions during appointments [38].

Most of the studies underscored the crucial role of patients’ regular access to and use of EHRs in enhancing the frequency and quality of communication between patients and HCPs concerning disease monitoring. Patients perceive digital health tools integrated with EHRs, such as videos, checklists, and SMS text messaging, as valuable contributors to fostering communication with their health care teams [36]. The delivery of visit notes electronically through OpenNotes further contributed to enriched communication [38].

Access to EHRs not only enhances communication but also facilitates collaborative discussions about patient treatment plans. Visualized data play a pivotal role in this dimension, emphasizing the value of data presentation in health care discussions [32]. The collaborative nature of discussions, particularly around medication reconciliation and barriers to use, is highlighted as vital for improved communication [27,30,38]. Shared decision-making emerged as a significant outcome influenced by patient access to EHRs. The use of a web-based coaching program using EHRs contributes to the sharing of patients’ medical information among HCPs, promoting dynamic management and follow-up analysis of patients’ diseases [26]. The seamless sharing of life log data through EHR-tethered PHR applications supports collaborative decision-making [33]. Patient access to the MHV program, for instance, reveals a preference for information sharing, emphasizing the collaborative nature of health care decisions [34]. In addition, access to PCEHR empowers patients to make informed choices about their health care [35].

The studies underscore that trust in HCPs and the overall patient-HCP relationship are positively influenced by patient access to EHRs [31,32,36]. Improved communication, particularly discussions centered on visualized data, contributes to the enhanced relationship between patients and physicians [32]. The heightened trust in HCPs is also evident through patient access to EMRs [31,36].

Patient satisfaction and health outcomes represent a critical dimension in understanding the impact of patient access to EHRs. Across the 18 studies included in this review, several dimensions emerged, reflecting the diverse effects on patients’ satisfaction and overall health. A notable finding suggests that patient access to EHRs contributes to perceived effectiveness in treatments [26,32,33,40]. Patients reported an enhancement in the quality of their treatment, as seen in the positive health outcomes associated with interventions such as web-based coaching programs [26]. However, variations in effectiveness were observed, as seen in the study by Wagner et al [40], where no impact on blood pressure was observed in the overall cohort, but active PHR users showed a significant reduction in diastolic blood pressure.

Similarly, several studies highlighted the improvement in health conditions and the quality of health care and life through patient access to EHRs [26,27,31,35]. Access through different platforms, including web-based coaching programs and patient portals, demonstrated that positive impacts on patient care resulted from treatment adherence and communication with HCPs [27]. However, it is crucial to note that not all interventions demonstrated uniform positive effects. For instance, the study by Wagner et al [40] found no impact of PHRs on blood pressure. Furthermore, some studies emphasized the significance of functions tailored to personalized preferences [25,34,42]. Patient portals positively influenced health outcomes by providing personalized reminders for appointments and screenings [25]. The IPHR showcased its capability to offer personalized recommendations, and usability tests on PHRs provided insights into the importance of tailoring functions to individual preferences [34,42].

Two studies highlighted the achievement of health-related goals through patient access to EHRs [34,42]. The IPHR effectively engaged patients in preventive care, conducting a concise health risk assessment that considers various aspects of patients’ daily lives, including physical, mental, and social well-being [42]. The MHV program, integrated into PHRs, also facilitated patients in adapting to specific health-related goals [34]. Furthermore, notably, the prompt delivery of results through web-based access to laboratory test results contributed to higher satisfaction levels among service or EHRs users [39].

One study indicated that accessing EHRs through patient portals positively impacted the effective use of health care resources by extending doctor’s visits, which ultimately improved the effectiveness of interactions with HCPs [25]. This suggests that patient access to EHRs may play a role in optimizing the use of health care resources during medical appointments. Furthermore, efficient use of health care and information resources is identified as another dimension influenced by patient access to EHRs.

A study by Krist et al [42] demonstrated that the IPHR, using EMRs, established an open database connection to link with the EMR of the patient’s designated clinician. This connection enabled patients to access critical clinical data, including medical history, medications, immunizations, and test dates. The platform empowered patients to actively engage in the review, correction, and updating of their health information as needed. This emphasizes the potential of patient access to EHRs in achieving efficient use of health care and information resources.

Focusing on the use of preventive care services, Krist et al [42] highlighted that, through the use of EMRs and the IPHR, patients were effectively engaged in preventive care. This involvement enabled patient access to results related to preventive care, with a use rate ranging from 1.5% to 28.3% across different health care practices. This suggests that patient access to EHRs may contribute to increased participation in preventive care services, promoting a proactive approach to health care management.

Notably, given the focus of the included studies on the use of health information resources, there is a lack of studies addressing the effectiveness of health care resource use and adherence to follow-up appointments, addressing this nuanced aspect of patient engagement that requires further exploration.

Importantly, some of the included studies highlighted some usability concerns and barriers related to patient access to and use of EHRs. One study emphasized the need for PHR registration to strike a balance between simplicity, security, and privacy [34]. Participants using EHRs via patient portals expressed concerns about the potential damage or hacking of their web-based records [25]. Technical barriers were also reported in 3 studies [25,28,30]. Consumers expressed apprehensions about obstacles to use, including intricate visual layouts and suboptimal usability features [25]. Patient access to documents in health records through the patient portal was considered intricate [28].

Usability challenges encompassed EHR integration, concerns with the EHR-portal communication interface, and issues related to browser access and compatibility, affecting both patients and HCPs [30]. In addition, barriers in the comprehension of health information and medical terms were reported in several studies [25,28,29]. Consumers expressed concerns about the complexity of language used in EHRs [25]. Challenges included patients grappling with medical terminology and the necessity for rigorous inspection of medical documents [28,29], which hindered their engagement with health care services. One study [39] demonstrated that there was no notable distinction between the groups (comprising those with web-based access to laboratory test results via EHRs and the no access control group) concerning the levels of anxiety reported following the receipt of test results through EHRs.

All these EHR usability–related concerns align with the 8 reasons identified by Valeur et al [47] for participants’ hesitance to embrace patient-accessible EHRs. Patients expressed a variety of concerns, including finding PAEHRs unnecessary; preferring oral communication with HCPs; difficulty understanding the medical terminology in the records; dissatisfaction with the records’ focus solely on disease; concerns about emotional reactions; perceiving it as an additional burden on their role as a patient; feeling that it was cumbersome, particularly among those lacking digital competence; and expressing skepticism about the overall impact of digital transformation on individual and social life.

As a result, there is a scarcity of research examining the possibility that EHR access could heighten feelings of uncertainty and anxiety when patients encounter clinical information that is unclear, particularly in the context of severe clinical conditions [5]. However, it is noteworthy that such an impact was not observed in the studies included in our analysis.

Interestingly, the diverse array of study designs, settings, and sample demographics observed across the 18 included studies underscores the multifaceted nature of patient engagement research, providing an excellent opportunity to highlight gaps in the existing literature and emphasize the unique contribution of our study. While observational cross-sectional studies dominate the literature, revealing prevalent trends, they inherently lack the capacity to establish causation over time [32,34,37]. By contrast, the presence of RCTs and mixed methods designs showcases the field’s commitment to rigorous investigation, yet limitations such as ethical concerns and challenges in real-world applicability persist [26,30,33,34,36,42]. The inclusion of case reports, while offering unique insights, raises questions about generalizability [38].

Regarding the reliability of the positive impact of EHRs on patient engagement that was reported in most of the included studies, the critical evaluation of this evidence reveals a spectrum of strength influenced by study designs, sample sizes, and primary outcome measures. Robust evidence emerged from large-scale RCTs [26,33,40], nonrandomized [31,39,41], or descriptive studies [27,32,37,38] with large number of participants, where patient engagement serves as the primary outcome. These studies offer statistically significant insights, enhancing the reliability of reported outcomes. In contrast, qualitative studies [25,28,35], though rich in depth, exhibit a lower strength of evidence due to smaller sample sizes and exploratory nature. Furthermore, the variability in methodologies and the potential for publication bias, which were acknowledged in some studies [34,35], necessitate a nuanced interpretation.

Despite demographic diversity, with variations in age, sex, and education levels, certain populations remain underexplored, and the need for targeted interventions in EHRs access areas becomes evident [25-42]. Moreover, the diverse clinical settings, from dermatological outpatient clinics to primary care practices and academic medical centers, highlight the variability in contextual factors influencing patient engagement interventions [28,32,34,42]. Identified gaps include the limited representation of specific age groups or cultural backgrounds, a scarcity of longitudinal studies to assess sustained effects, and a dearth of real-world implementation studies. In addressing these gaps, our study contributes novel insights and practical solutions for advancing patient engagement research related to access to patient EHRs. By examining various types, designs, settings, and demographics in our included studies, we offer a more comprehensive understanding of EHR effectiveness for patient health care engagement across diverse health care settings.

It is important to note the “supply side” of patient engagement issues because the success or failure of this engagement depends on the role played by HCPs, HIT teams, and health systems regarding EHRs. The implementation and support of EHRs present multifaceted challenges for these parties. HCPs contend with an onerous administrative burden, grappling with the time-consuming demands of data entry, documentation requirements, and regulatory compliance. Concurrently, HIT teams navigate technical complexities, encompassing software customization, data security, and interoperability, amidst an ever-evolving landscape of health care technology. Resource constraints within health systems, including financial limitations and staffing shortages, further exacerbate the challenges of investing in EHR infrastructure, providing adequate training, and addressing emerging implementation issues. Compounding these challenges is user resistance, as HCPs and staff may exhibit skepticism toward the benefits of EHRs and apprehension about workflow disruptions. Moreover, achieving seamless interoperability and data exchange between different EHR systems remains challenging due to incompatible standards, data silos, and privacy concerns. Addressing and actively mitigating these challenges necessitates a collaborative effort among health care stakeholders, encompassing policy makers, health care organizations, technology vendors, and clinicians for effective EHR implementation and support, thus enhancing patient health care engagement and delivery.

In addition, administrative burden relative to patient portals and EHRs is different among different health systems. Many studies included in this systematic review are from countries with national health systems. The clinical workflows and administrative burdens in these countries can differ significantly from those in the United States, where most included studies (8/18, 44%) were conducted. In the United States, HCPs often face a high administrative burden due to extensive documentation requirements, data entry, and regulatory compliance. This burden is exacerbated by the complexity of the US health care system, which involves multiple payers and fragmented care delivery.

In contrast, several studies come from countries with national health systems, including Sweden, Canada, Australia, and the Netherlands. These countries often experience more streamlined administrative processes. For example, Sweden’s national health system emphasizes integrated care and standardized EHR systems, which can reduce the administrative burden on clinicians. Similarly, Canada’s single-payer system and Australia’s national health infrastructure support more cohesive HIT integration, easing the documentation load for HCPs.

Overall, while the administrative burden associated with EHRs and patient portals can be substantial in the United States, it may be lower in countries with more integrated and streamlined HIT processes, national-level support, and fewer regulatory complexities. Addressing these differences is crucial for understanding the global implementation and impact of EHRs and patient portals on health care engagement.

In addition, the use of EHRs is influenced by cultural practices and health care resources across diverse health care settings. Cultural norms regarding privacy, patient autonomy, and technology shape patient engagement with EHRs, emphasizing the need for culturally aligned systems. Disparities in health care resources, including technological access, contribute to variations in EHR availability, underscoring the importance of equitable access. In addition, practice cultures influence EHR adoption, with collaboration and continuous learning essential for optimizing outcomes. Addressing these factors can enhance EHR use and health care engagement globally.

### Strengths of the Study

This study exhibits several notable strengths that contribute to the robustness and reliability of its findings. First, the comprehensive nature of our systematic review, which included 18 diverse studies addressing diverse EHRS-related applications and platforms and using various methodologies or study types (quantitative, qualitative, and mixed methods), ensures a thorough exploration of patient engagement in the context of EHRs. In addition, the inclusion of different study designs (ranging from observational cross-sectional studies to RCTs) enriches the depth and breadth of our analysis. Furthermore, our study meticulously examined the variations in patient demographics across these diverse studies, providing valuable insights into the generalizability of findings across different populations. The systematic approach used in our review, adhering to established protocols and guidelines (ie, PRISMA) enhances the transparency and validity of the synthesis. In addition, by identifying gaps and variations in the existing literature, our study sets the stage for future research endeavors to address these nuances and contribute to the evolving field of patient engagement with EHRs.

Moreover, our research distinguishes itself by providing a more comprehensive array of outcome measures for patient health care engagement. Unlike the included studies, which predominantly offered 1 or 2 measures, our study adopts a more holistic approach. By encompassing a broader spectrum of outcomes, we aim to capture the multifaceted nature of patient engagement, contributing a nuanced perspective to the existing body of literature. This approach ensures a more thorough understanding of the impact of patient access to EHRs on diverse facets of health care engagement.

### Limitations of the Study

It is crucial to acknowledge certain constraints inherent in this systematic review. Publication bias may influence the results, with a potential inclination toward publishing studies with positive outcomes. The diverse methodologies and designs among the included studies introduce variability in evidence quality, challenging uniform analysis. Constraints include the limited availability of studies focusing on specific dimensions of patient engagement, impacting the review’s comprehensiveness. Variations in sample sizes, demographics, and clinical settings may also affect the generalizability of findings. In addition, the dynamic nature of health care systems and technological advancements could render some findings time sensitive. Moreover, the use of 4 databases, while relevant to health care publications, may introduce the inadvertent inclusion of unrelated studies. Despite a comprehensive search strategy, PsycINFO yielded a limited number of results (n=18), potentially influencing the diversity of included studies and impacting the overall synthesis. These limitations underscore the importance of interpreting findings with consideration for the scope and potential variations in search outcomes.

Importantly, while the included studies provided valuable insights into the impact of interventions involving patient access to EHRs on patient engagement, it is important to acknowledge a limitation inherent in the design of these interventions. Many of the identified studies encompass multifaceted interventions, incorporating various components alongside patient access to EHRs. The holistic evaluation of these interventions poses a challenge in isolating and quantifying the distinct contribution of EHR access to observed improvements in patient engagement. This limitation implies that the specific impact of patient access to EHRs, when considered in isolation, remains unclear.

### Implications for Future Research

#### Clinical Implications

The findings of this systematic review carry significant clinical implications for health care practitioners and HCPs. Understanding the positive impact of patient engagement with EHRs on various dimensions, including improved adherence to treatment plans, enhanced communication, and increased patient satisfaction, underscores the potential for integrating EHRs as a crucial component of patient care. Clinicians can leverage these insights to encourage and facilitate patient access to EHRs, promoting active involvement in their health care journey. Efforts to enhance user-friendly interfaces and address privacy concerns can further optimize the clinical utility of EHRs. Moreover, the identified gaps in the literature suggest avenues for targeted interventions to address specific dimensions of patient engagement that may currently be underexplored in clinical practice.

The clinical implications may extend to specific patient populations and health care settings, such as patients with long-term conditions and chronic disease in primary care in the United Kingdom, emphasizing the substantial potential for continuity of care and enhanced outcomes through EHR accessibility. Furthermore, patient use of EHRs can benefit the health care system by contributing to alleviating the economic impact of chronic diseases by efficiently managing population health within the community. Therefore, EHRs presents an optimal platform for proactive engagement for both HCPs and patients. In addition, in universal health coverage systems, patient access to primary care EHRs contributes to a more holistic approach to health care. It is crucial to recognize that EHR accessibility requires careful consideration to avoid potential harms related to information exchange, emphasizing the need for a robust electronic bioinformatic initiative scheme.

#### Research Implications

The systematic review reveals several research implications that can guide future investigations in the realm of patient engagement with EHRs. The identified gaps in the literature, such as the limited focus on certain dimensions of patient engagement and variations in study methodologies, highlight areas where further research is warranted. Future studies should aim for more standardized approaches to enable meaningful comparisons across interventions and settings. In addition, the potential publication bias and time-sensitive nature of findings emphasize the need for continuous updates and assessments to capture the evolving landscape of health care technology. Researchers are encouraged to delve deeper into specific dimensions, populations, and health care settings that may not have been extensively studied. Advancements in technology and changes in health care delivery models should be taken into account to ensure the relevance and applicability of future research in enhancing patient engagement with EHRs.

### Conclusions

This systematic review provides a comprehensive synthesis of existing literature on patient engagement with EHRs. The findings highlight the positive impact of EHR accessibility on various dimensions, including increased adherence to treatment plans, promoted involvement and empowerment, improved communication, and enhanced patient satisfaction. While acknowledging the limitations and variations in study methodologies, this review underscores the importance of EHRs as a valuable tool in promoting patient engagement and active participation in health care. The identified gaps in the literature present opportunities for future research to explore specific dimensions of patient engagement and optimize the clinical utility of EHRs. Overall, this study contributes to the evolving understanding of the role of EHRs in patient health care engagement, emphasizing the need for continued exploration and refinement in this critical area of health care delivery.

## Appendix

Multimedia Appendix 1 PRISMA (Preferred Reporting Items for Systematic Reviews and Meta-Analyses) checklist.

Multimedia Appendix 2 Results of the search strategies used in Ovid (MEDLINE), Ovid (Embase), Ovid (PsycINFO), and EBSCOhost (CINAHL).

Multimedia Appendix 3 The characteristics of the selected studies.

Multimedia Appendix 4 Quality assessment of the included studies using the Mixed Methods Appraisal Tool.

## Abbreviations

COPD: chronic obstructive pulmonary disease
EHR: electronic health record
EMR: electronic medical record
HCP: health care provider
HIT: health information technology
IPHR: interactive patient health record
MHV: MyHealtheVet
MMAT: Mixed Methods Appraisal Tool
PCEHR: personally controlled electronic health record
PHR: personal health record
PICO: population, intervention, comparator, and outcome
PRISMA: Preferred Reporting Items for Systematic Reviews and Meta-Analyses
RCT: randomized controlled trial
