# Supplementary material for: The Impact of Patient Access to Electronic Health Records on Health Care Engagement: Systematic Review
Source: J Med Internet Res. 2024 Nov 20;26:e56473. doi: 10.2196/56473 (PMC11618012; doi:10.2196/56473)
Supplement: Multimedia Appendix 2 [file jmir_v26i1e56473_app2.doc]

**Multimedia Appendix 2: Results of the search strategies used in Ovid (MEDLINE), Ovid (Embase), Ovid (PsycINFO), and EbScohost (CINAHL)***

| **Search strategy as used in Ovid MEDLINE(R), Embase & PsycINFO** |
| --- |
| **Part 1: Electronic health record**  1 Electronic Health Record*.mp. or EHR/  2 Electronic Medical Record*.mp. or EMR/  3 Electronic Hospital Record*.mp. or Computerized medical record/ or Automated medical record/  4 Personal Health Record*.mp. or PHR/  5 Personal Medical Record*.mp.  6 Patient Health Record*.mp.  7 Patient-Controlled adj3 Health Record*.mp.  8 Controlled Health adj3 Management System*.mp.  9 Mobile Apps Personal Health Record.mp.  10 Patient adj3 health self-management.mp. or Patient Care Management/  11 Telehealth Record*.mp.  12 Patient Generated adj3 Health Data.mp. or Personal health information/  [mp=title, book title, abstract, original title, name of substance word, subject heading word, floating sub-heading word, keyword heading word, organism supplementary concept word, protocol supplementary concept word, rare disease supplementary concept word, unique identifier, synonyms, population supplementary concept word, anatomy supplementary concept word]  **13 1 or 2 or 3 or 4 or 5 or 6 or 7 or 8 or 9 or 10 or 11 or 12 (Combined with ‘OR’ operator)**  **Part 2: Patient access**  14 Access*.mp.  15 Usage.mp.  16 Practice.mp.  17 Adopt*.mp.  18 Utiliz*.mp.  19 Utilis*.mp.  20 Employ*.mp.  21 Implement*.mp. or organization and management/  22 Entry.mp.  23 Retrieve*.mp. or [information retrieval](https://ovidsp-dc1-ovid-com.manchester.idm.oclc.org/ovid-new-a/ovidweb.cgi?&Controlled+Vocabulary=Mapping|2&Return=mapping&S=GCAJFPIKPGACNMAJKPKJPEOIIJMCAA00)/  24 Review.mp. or medical record review/  [mp=title, book title, abstract, original title, name of substance word, subject heading word, floating sub-heading word, keyword heading word, organism supplementary concept word, protocol supplementary concept word, rare disease supplementary concept word, unique identifier, synonyms, population supplementary concept word, anatomy supplementary concept word]  **25 14 or 15 or 16 or 17 or 18 or 19 or 20 or 21 or 22 or 23 or 24 (Combined with ‘OR’ operator)**  **Part 3: Healthcare engagement**  26 Patient* adj3 Engage*.mp.  27 Patient* adj3 Participat*.mp.  28 Patient* adj3 Involve*.mp.  29 Patient* Activation.mp.  30 Patient* adj3 Empower*.mp.  31 Patient* adj3 Communicat*.mp.  32 Healthcare adj3 Engage*.mp.  33 Health care adj3 Engage*.mp.  34 Patient* Enrollment*.mp.  35 Patient* Awareness.mp.  36 Personal Health ADJ3 Enrollment.mp.  37 Patient Acceptance.mp. or Patient Acceptance of Health Care/  38 Functionalities.mp.  39 Interoperability.mp.  40 Patient Uptake.mp.  41 Patient Incorporation.mp.  42 Patient Attitude.mp. or Health Knowledge, Attitudes, Practice/ or Attitude to Health/  43 Patient* Experience.mp.  44 Patient* Interaction.mp.  45 Patient* Perception.mp.  46 User* Experience.mp.  47 Medical Communication.mp.  48 Emergency Medical Services.mp.  49 Health Information Exchange.mp.  50 Mutual understanding.mp.  51 Shared Decision making.mp. or Decision making, Shared/  52 Patient*-provider* relation*.mp.  53 Patient*-provider communication.mp.  54 Physician*-patient* relation*.mp.  55 Physician*-patient* communication*.mp.  56 Doctor*- Patient* communication.mp.  57 Doctor*- Patient* relation*.mp.  58 Informed patient communication  59 Informed Consent or "Patient* risk communication.mp.  60 Patient *commitment.mp.  61 Patient* ADJ3 Instructions.mp.  62 Patient* readiness.mp.  63 Patient ADJ3 appointment*.mp. or Patient schedule*/  64 Patient* literacy.mp.  65 Health literacy.mp. or Health technology literacy/  66 Patient* adherence.mp. or Medication adherence/  67 Medication compliance.mp.  68 Patient health monitoring.mp.  69 Interactive patient.mp.  70 Second opinion.mp.  71 Proactive* medication.mp. or Preventive medication/  72 Medical advice*.mp.  73 Patient relocation.mp.  [mp=title, book title, abstract, original title, name of substance word, subject heading word, floating sub-heading word, keyword heading word, organism supplementary concept word, protocol supplementary concept word, rare disease supplementary concept word, unique identifier, synonyms, population supplementary concept word, anatomy supplementary concept word]  **74 26 or 27 or 28 or 29 or 30 or 31 or 32 or 33 or 34 or 35 or 36 or 37 or 38 or 39 or 40 or 41 or 42 or 43 or 44 or 45 or 46 or 47 or 48 or 49 or 50 or 51 or 52 or 53 or 54 or 55 or 56 or 57 or 58 or 59 or 60 or 61 or 62 or 63 or 64 or 65 or 66 or 67 or 68 or 69 or 70 or 71 or 72 or 73 (Combined with ‘OR’ operator)**  **75 13 and 25 and 74 (Final results combined with ‘AND’ operator)** |

| **Search strategy as used in Ebscohost (CINAHL PLUS)** |
| --- |
| **Part 1: Electronic health record**  S1 “Electronic Health Record*”  S2 “Electronic Medical Record*”  S3 “Electronic Hospital Record*”  S4 “Personal Health Record*”  S5 “Personal Medical Record*”  S6 “Patient Health Record*”  S7 Patient-Controlled N3 Health Record*  S8 Controlled Health N3 Management System*  S9 “Mobile Apps Personal Health Record”  S10 Patient N3 health self-management  S11 “Telehealth Record*”  S12 Patient Generated N3 Health Data  **S13 S1 OR S2 or S3 OR S4 OR S5 OR S6 OR S7 OR S8 OR S9 OR S10 OR S11 OR S12 (Combined with ‘OR’ operator)**  **Part 2: Patient access**  S14 Access*  S15 Usage  S16 Practice  S17 Adopt*  S18 Utiliz*  S19 Utilis*  S20 Employ*  S21 Implement*  S22 Entry  S23 Retrieve*  S24 Review  **S25 S14 OR S15 OR S16 OR S17 OR S18 OR S19 OR S20 OR S21 OR S22 OR S23 OR S24 (Combined with ‘OR’ operator)**  **Part 3: Healthcare engagement**  S26 Patient* N3 Engage*  S27 Patient* N3 Participat*  S28 Patient* N3 Involve*  S29 “Patient* Activation”  S30 Patient* N3 Empower*  S31 Patient* N3 Communicat*  S32 Healthcare N3 Engage*  S33 Health care N3 Engage*  S34 “Patient* Enrollment*”  S35 “Patient* Awareness”  S36 Personal Health N3 Enrollment.  S37 “Patient Acceptance”  S38 Functionalities  S39 Interoperability  S40 “Patient Uptake”  S41“Patient Incorporation”  S42 “Patient Attitude”  S43 “Patient* Experience”  S44 “Patient* Interaction”  S45 “Patient* Perception”  S46 “User* Experience”  S47 “Medical Communication”  S48 “Emergency Medical Services”  S49 “Health Information Exchange”  S50 “Mutual understanding”  S51 “Shared Decision making  S52 “Patient*-provider* relation*”  S53 “Patient*-provider communication”  S54 “Physician*-patient* relation*”  S55 “Physician*-patient* communication*”  S56 “Doctor*- Patient* communication”  S57 “Doctor*- Patient* relation*”  S58 “Informed patient communication”  S59 “Patient* risk communication”  S60 “Patient *commitment”  S61 Patient* N3 Instructions  S62 “Patient* readiness”  S63 Patient N3 appointment*  S64 “Patient* literacy”  S65 “Health literacy”  S66 “Medication adherence”  S67 “Medication compliance”  S68 “Patient health monitoring”  S69 “Interactive patient”  S70 “Second opinion”  S71 “Proactive* medication”  S72 “Medical advice*”  S73 “Patient relocation”  **S74 S26 or S27 or S28 or S29 or S30 or S31 or S32 or S33 or S34 or S35 or S36 or S37 or S38 or S39 or S40 or S41 or S42 or S43 or S44 or S45 or S46 or S47 or S48 or S49 or S50 or S51 or S52 or S53 or S54 or S55 or S56 or S57 or S58 or S59 or S60 or S61 or S62 or S63 or S64 or S65 or S66 or S67 or S68 or S69 or S70 or S71 or S72 or S73 (Combined with ‘OR’ operator)**  **S75 S13 and S25 and S74 (Final results combined with ‘AND’ operator)** |

***All search strategies were limited to the English language and publication of January 1, 2010 till 15 November, 2023**

***The search is done on 15 November, 2023**
