# Supplementary material for: The Impact of Patient Access to Electronic Health Records on Health Care Engagement: Systematic Review
Source: J Med Internet Res. 2024 Nov 20;26:e56473. doi: 10.2196/56473 (PMC11618012; doi:10.2196/56473)
Supplement: Multimedia Appendix 3 [file jmir_v26i1e56473_app3.doc]

|  | **Multimedia Appendix 3: The Characteristics of selected studies** | | | | | | | | |
| --- | --- | --- | --- | --- | --- | --- | --- | --- | --- |
| **No.** | **Author (s), year & country** | **Study aim/objective** | **Intervention** | **Study type/ design** | **Data collection method** | **Sample size and**  **demographics** | **Clinical/**  **study setting** | **Measures of patient engagement (outcome)** | **Main findings** |
|  |  |  |  |  |  |  |  |  |  |
| 1 | Zarcadoolas et al. (2013) [25]  United States (New York City) | Identify vulnerable consumers’ response to patient portals, their perceived utility and value, as well as their reactions to specific portal functions. | Patient portals | Focus groups  (Qualitative) | Focus groups | 28 participants (10 males and 18 females)  - Age range: 21-63 years  - New York City resident,  - able to read and speak English,  - no higher than a high school education/GED.- had experience with computers, and use the Internet. |  | - Consumer engagement/patient empowerment - Doctor’s visit/ communication with health care providers - Health literacy factors - Prevention and health maintenance - Privacy and security | - Patient portal use made consumers most positive about features that increased convenience (empowerment), such as making appointments, refilling prescriptions (medication management) - It extended doctor’s visit - It enhanced communication with HCPs. - Positive health impacts of reminders for appointments, annual visits, and screenings - Consumers raised concerns about a number of potential barriers to usage, such as complex language, complex visual layouts, and poor usability features. - Small number of participants voiced concerns related to the privacy and security of online records (damage, hacking). |
| 2 | Wang et al. (2017) [26]  China | Develop and evaluate a Web-based coaching program using EHRs for physical function and health-related quality of life for patients with Chronic obstructive pulmonary disease (COPD) in China | - Web-based coaching program using EHRs | Randomized Controlled Trial (RCT)  (Quantitative) | Questionnaires and clinical variables | - Total participants: 130 (120 participants completed the 12-month follow-up program)   Patients who were definitively diagnosed with COPD | Two hospitals in China | - Lung function measures | - The Web-based coaching program using EHRs in China appears useful for patients with COPD transitioning from the hospital to the community. - It promotes the sharing of patients’ medical information by hospital and community nurses, and achieves dynamic management and follow-up analysis for patients’ disease. - This program can postpone the decreasing rate of lung function, improve quality of life, decrease dyspnea, and increase physical capacity |
| 3 | Nazi et al. (2013) [27]  United States | Explore Veteran perceptions about access to their medical records through a PHR tethered to an EHR, including perceived value and effect on satisfaction, self-care, and communication. | - My HealtheVet Pilot Program, a PHR prototype enabling patients to import 18 types of information, including clinical notes and laboratory test results, from the VA EHR into a secure PHR portal. | Cross-sectional study  (Quantitative) | Web-based survey | 688 Veteran respondents (Veterans enrolled in the My HealtheVet Pilot Program)  Intermediate and high level of internet skills | Department of Veteran Affairs (VA) | - Satisfaction with the pilot program, - Perceived helpfulness of information and services, - Ease of locating relevant information, - Perceived improvement in care, - Likelihood to recommend the program | - Improved enrollment (most Veterans reported that they either had received no training about using the pilot Web site (43%) or used self-instruction materials). - High satisfaction with the pilot program (84% finding the information and services helpful). It improve their understanding of their medical problems and treatments - Feature Utilization: The majority (72%) indicated that the pilot website made it easy to locate relevant information (using the health education library, self-entering data using the Web site’s Healthelog features, looked up information on a disease, condition, or treatment, and discussed information with their health care provider). - The most useful features were viewing portions of their medical record, accessing their prescription history, and viewing appointments - Most participants agreed that it helped improve their care (Wellness Reminders had helped them to take action, stick to their treatment plan, improved communication with their health care provider), with 90% indicating they would recommend it to another Veteran. |
| 4 | Suija et al. (2022) [28]  Estonia | - Investigate the experiences of patients and primary care physicians with health data, including patient-generated and physician-generated data during the COVID-19 pandemic   Explore expectations and obstacles encountered in using health data. | - Patient access to health record with using its health data | (Qualitative) | Individual semi-structured interviews with patients and physicians | 14 (7 Adults patients, 7 physicians) | Primary care settings | - Patient and physician perspectives on the importance of easy access to health data. - Challenges related to medical terminology. - Views on patient-generated data as a valuable information source. | - - Both patients and physicians emphasized the importance of easy access to health data in digital health information systems.   - Challenges identified include patients' understanding of medical terminology and the need for quality inspection of medical documents.   - Agreement on the usefulness of patient-generated data and a call for increased utilization - **Three main themes:**  1. **Access to health records:**  - Patient found that it is important to access to health records (hospital epicrisis, consultation answers, investigation results, etc.) but access to these documents via the Patient Portal is complicated - Physicians found it is important that all health data is consolidated in the central health information system, which functions efficiently and is also secure. - Physicians reported that easy access to previous patient health records is important to be able to prepare a complete treatment plan.   **2) Experience with using data in health records:**   - Patients mentioned that they expect medical records to give more information about disease, in general, as well as instructions for further self-care. - Physicians were concerned about the inconsistent quality of medical papers (e.g. hospital epicrises, which sometimes only presented laboratory findings, but not a synthesis of the information and depend on the physician) as well as a clear management plan for the follow-up period (e.g. who is responsible for what) is often missing from the medical documents.   **3) The use of patient-generated data.**   - Patient found that they were ready to generate important data required for medical decisions (questionnaires filled in before the appointment could help to systematize complaints and also save time during the consultation). - Physicians found that patient generated data is valuable, and they took it into account when making treatment decisions. |
| 5 | Wass et al. (2019) [29]  Sweden (Jönköpin) | - Explore how patients perceive the possibility of accessing their EHRs online.   Investigate whether online access to EHRs influences patient involvement | - Online access to EHRs via the patient portal | Mixed method (quantitative and qualitative | Interviews and a survey | - 9 patients (Interviews) - 56 patients (Survey) - 4 women and 5 men, age(34 - 83 years), and all had accessed EHRs during the study. | - The county hospital   The survey at (Sweden: a primary care unit, a cardiology outpatient clinic and a urology outpatient clinic) | - Accessibility of healthcare information through EHRs. - Recall, understanding, and patient involvement | - Patients perceive improved accessibility of healthcare information through EHRs (the access facilitated the understanding, discussing, coordinating) of what was documented. - EHR accessibility is associated with enhanced patient involvement in their treatment (follow the advice given by HCPs, recall, easier to take responsibility for their care, easier to understand what was said during the appointment, easier to talk to healthcare professionals about their situation) - Only 9% worried or upset about the information in the EHRs, some of the information in the EHR was difficult to understand as it include medical terms. |
| 6 | Spratt et al. (2022) [30]  United States | - Evaluate the Duke PillBox application, a SMART-on-FHIR medication management application integrated into the EHRs patient portal.  - Assess usability and feasibility of the Duke PillBox application for patients with diabetes and uncontrolled hypertension. | - The Duke PillBox application integrated into the EHRs patient portal | Prospective, mixed-methods (pre-post implementation design)  (Quantitative & Qualitative) | Usability/feasibility survey.  Communication with a pharmacist via the portal-based application.    - Medication reconciliation and discussion of medication indications, side effects, and barriers to use with patients | 285 eligible patients (of 29 interested and only 12 participated).   - Adults with active portal status, diabetes, and uncontrolled hypertension | Academic health system | - Activities accessed and viewed within MyChart. - Messages exchanged with providers. - Number of visits to the health system - Technology-savviness - patient interactions (Medication adherence, Provider communicatio) - Patient education - Feedback on the patient experience (Challenges to usability). | - The Duke PillBox application is desirable based on improved patient interactions (patient took medication as prescribed, improved health knowledge) - Challenges to usability include EHRs integration and issues with the EHR-portal communication interface, browser access and compatibility for both patients and providers. |
| 7 | van Der Vaart et al. (2014) [31]  Netherlands | Measure the use, satisfaction, and impact of a web portal providing patients with rheumatoid arthritis home access to their electronic medical records (EMR). | - Hospital-based patient web portal providing home access to EMR | Pretest-posttest study  (Quantitative) | Questionnaires | 360 patients. | Hospital-based patient web portal. | - Self-efficacy in patient-provider communication. - Patient-provider relationship (trust in their rheumatologist - Patient empowerment (Illness perceptions, Medication adherence, involvement in treatment and knowledge about treatment) - Patient satisfaction with care. | - 54% of respondents with Internet access viewed their EMR. - Respondents were positive about the ease of use and usefulness of the portal with very few reported problems. - Age, amount of Internet use, and self-perceived Internet skills significantly predicted portal use. - Of those who logged in, 44% felt more involved in their treatment, and 37% felt they had more knowledge about their diseases and treatment. - The quality of care is higher after using portal. - Self-efficacy in patient-provider Communication as well as the trust in them increased. - Increased medication adherence |
| 8 | Klein et al. (2020) [32]  Germany | Investigate and compare patients' and physicians' attitudes towards using (EHRs) in routine psoriasis care, with a focus on assessing their acceptability and expectations regarding potential EHR uses. | Use of EHRs in psoriasis care | Observational cross-sectional  (Quantitative) | Paper-based or an electronic version of the questionnaire. | 187 patients and 44 dermatologists  Patient Sample: 187 patients, mean age 51.62 years, 51.4% men.  Physician Sample:  44 dermatologists, mean age 53.30 years, 68.2% men. | Dermatological outpatient clinic and via online pathways, including patient associations and social media | - Monitoring disease - Communication and relationship between patients and physicians - Treatment quality - Compare visualized patient data between each other | - Both patients and physicians considered it is important for physicians to retrieve data quickly - For patients, by using an EHR, the course of the disease can be monitored longitudinally. - The communication and the relationship between patients and physicians can improve based on patient access to and discussion based on visualized data. - Reduced physicians workload due to the use of an EHRs. - Patients expected more often that the use of an EHRS can improve quality of the treatment. - Patients found it interesting to compare their own data with data of other patients, allowing them discuss more informed with their physicians. - Physicians concerns about the feasibility of having enough time for EHR data and patients consciously maintaining EHR data. |
| 9 | Ryu et al. (2017) [33]  Korea | - Demonstrate the development of an electronic health record (EHR)–tethered PHR app named MyHealthKeeper.   Study the effectiveness of a PHR data-driven clinical intervention with clinical trial results | -MyHealthKeeper PHR app   - PHR-based clinical intervention | Clinical trial with a 4-week study period (RCT)  (Quantitative) | Paper-based survey, laboratory test, physical examination, and opinion interview.  Use of a commercially available activity tracker (Misfit) to collect individual physical activity data.  - MyHealthKeeper mobile phone app to record participants’ patterns of daily food intake and activity logs. | 80 (51 in the PHR-based intervention group, 29 in the control group).  Completed the study: 68 (44 in the intervention group, 24 in the control group). | Seoul National University Bundang Hospital (SNUBH; Seongnam, Republic of Korea) | - Health outcome measures (weight change, changes in blood biochemical parameters (cholesterol, triglycerides, high-density lipoprotein cholesterol, and low-density lipoprotein cholesterol)) | - Developed an innovative EHR-tethered PHR system allowing clinicians and patients to share life log data. - Demonstrated the effectiveness of a patient-managed and clinician-guided health tracker system. - Improved patient clinical profiles, as evidenced by weight loss and lower triglyceride levels. |
| 10 | Haggstrom et al. (2011) [34]  United States | - Create user-centered design information to guide the development of personal health records (PHRs). | Usability assessments of VA’s MyHealtheVet (MHV) program | Mixed method (Quantitative and Qualitative) | Survey (efﬁciency measures) and Observational videos | 24 Veterans Administration (VA) patients who had not previously used MHV  22 males  2 females  average age 55 years | Three different primary care clinics at a VA medical center | - Efficiency in prescription refills. - Information sharing with their doctors. - Using functions of MyHealtheVet | - PHR registration must balance simplicity and security. - Usability tests guide how PHRs can tailor functions to individual preferences. - PHRs add value to users’ data by making information more accessible and understandable. - MHV enables users to track their own health with multiple types of self-reported health information, including vital signs, labs, tests, and food and activity journals. - Most participants preferred information sharing with their providers. - MHV facilitate searching for medical information (about post-traumatic stress disorder and healthy eating) |
| 11 | Hanna et al. (2017) [35]  Australia | - Explore patients’ experiences and perspectives of using a locally developed personally controlled electronic health record (PCEHR) implemented in an Australian health service. | Use of a (PCEHR) (Medenote) | Qualitative descriptive study | Individual semi-structured telephone interviews. | 12 Barwon Health patients (active users of / registered with the PCEHR) | Barwon Health (Australian health service) | - Quality of healthcare - Patient capacity for self-management | - Participants emphasized the advantages of PCEHRs, including improved quality of healthcare through improved communication. - Enhanced Increased patient agency (patient capacity for self-management, make independent, informed choices about their health) - It enabled direct and instant access to their health information without having to negotiate that access through a healthcare provider gatekeeper. Participants could use this information in conjunction with healthcare providers to make choices about their healthcare. - The PCEHR was seen as a convenient, secure, permanent storage facility and memory aid that enabled participants to review and self-monitor their health status and treatment. |
| 12 | Fuller et al. (2020) [36]  United States (Boston) | - Evaluate the implementation of a suite of digital health tools integrated with the EHR to engage hospitalized patients, caregivers, and their care team in preparing for discharge | Digital health tools integrated with the EHR, including a discharge video, checklist, and post-discharge text messaging | - Utilized the Reach, Effectiveness, Adoption, Implementation, and Maintenance (RE-AIM) framework.  (Mixed methods) | Survey and interviews (for Patient) and  focus groups (for Clinician) | 510 patients or caregivers | A large academic medical center (Brigham and Women’s Hospital) | - Participation rates in watching the video, completing checklists, and requesting post-discharge text messaging. - Concerns reported per checklist submission. - Patient and clinician perceptions | - Most patient survey participants perceived that the intervention promoted self-management and communication with their care team, thus confidence. - Third patients requested post-discharge text messaging. - Patient Concerns reported most commonly about medications and follow-up. - Clinicians identified challenges in awareness, workflow, technical optimization, and inconsistent leadership. |
| 13 | Moll et al. (2018) [37]  Sweden | - Investigate patients’ experiences of accessing their EHRs through the Swedish national patient portal, with a focus on describing user characteristics, usage, and attitudes toward the system. | Patient access to electronic health records (EHRs) through the Swedish national patient portal, specifically the PAEHR system called Journalen. | Quantitative | Survey (questionnaire) | 2587 users of Journalen  Majority are women and higher education level | Swedish national patient portal, specifically the PAEHR system called Journalen. | - Health status   - - Information to access (lab results) | - - There is a strong positive attitude toward using PAEHRs (Journalen)   - Improved patient empowerment, involvement, and security.   - Access to test results is perceived to be the most important category and log list the least important.   - It provides informed information about immunizations, test results, and visit history   - It makes them feel more informed; it improves communication between medical staff and them; it improves the understanding of their condition; and it makes them feel safe. - The majority of respondents have not been worried by the contents of Journalen. |
| 14 | Wolff et al. (2017) [38]  United States (Pennsylvania) | - Examine the acceptability and effects of delivering doctors’ visit notes electronically (via OpenNotes) to patients and care partners with authorized access to patients’ electronic medical records. | Delivery of doctors’ visit notes electronically via OpenNotes to patients and care partners with authorized access | Case report  (Quantitative) | Surveys administered at baseline and after 12 months of exposure to OpenNotes. | Adult patients (323 completed survey and 184 responded at follow up) and care partners (389 survey and 252 follow up) who shared access to their patient portal account. | Geisinger Health System. | - Agreement between patients and care partners about patient treatment plans - Discussions between them about patient care. - Patient confidence in managing their health. - Patient readiness to office visits - Patient understanding of their care   Communication between patients and provider | - Patients and care partners reported better agreement about patient treatment plans and more productive discussions. - Patients showed increased confidence in managing their health. - Patients felt better prepared for office visits. - Patients reported better understanding of their care. - Care partners were more likely to access and use patient portal functionality.   - Care partners reported improved communication with patients’ providers. |
| 15 | Mák et al. (2015) [39]  British Columbi (Canada) | - Increase understanding of how Web-based access to laboratory test results in British Columbia affects patients’ experiences. | Web-based access to laboratory test results | Exploratory study with a retrospective cohort design  (Quantitative) | Survey | - Service users (Web-based access): n=2047.   Comparison group (no Web-based access): n=1245. | Laboratory test results | - Reported wait-time for results. - test result comprehension - Anxiety levels after receiving test results. | - The majority of service users received their results in shorter time (within "a few days) compared to just over a third of the comparison group, thus they are satisfied with the service. - While most in both groups reported understanding/ comprehension their test results, the rate was slightly lower for service users (75.55% vs. 84.69%). - There was no significant difference between groups in levels of reported anxiety after receiving test results. |
| 16 | Wagner et al. (2012) [40]  United States | - Examine the impact of a personal health record (PHR) in patients with hypertension measured by changes in biological outcomes, patient empowerment, patient perception of quality of care, and use of medical services. | A PHR tethered to the patient’s electronic medical record (EMR) | Cluster-randomized effectiveness trial with PHR and no PHR groups  (Quantitative) | Internet Accessibility Questionnaire | 453 patients   - (with 55% classiﬁed as having metabolic syndrome. At study enrollment, average SBP was 131.4mm Hg and average DBP was 79.0mm Hg, with 30% having uncontrolled hypertension) | Two ambulatory clinics | - Changes in biological outcomes (e.g., blood pressure (BP). - Patient empowerment (measured by 13-item Patient Activation Measure and Patient Empowerment Scale) - Patient perception of quality of care (measured by both Consumer Assessment of Healthcare Providers and Systems & Patient Assessment of Chronic Illness Care), - Utilization measures (self-reported use of medical services). | - No impact of the PHR was observed on BP, patient activation, patient perceived quality, or medical utilization in the intention-to-treat analysis. - A sub-analysis of intervention patients who self-identified as active PHR users showed a 5.25-point reduction in diastolic BP. - Few patients provided with a PHR actually used it with any frequency. Younger age, self-reported computer skills, and more positive provider communication ratings were associated with frequency of PHR use. |
| 17 | Ibrahim et al. (2022) [41]  Malaysia (Seremban district) | - Compare the level of patient satisfaction between EMR-based clinics and paper-based clinics in Malaysian primary healthcare. | Implementation of the Teleprimary Care—Oral Health Clinical Information System (TPC-OHCIS), an updated electronic medical record (EMR) in selected clinics. | Quasi-experimental posttest design with a control group  (Quantitative) | Short-Form Patient Satisfaction Questionnaire with 7 subscales, | A total of 321 patients (with 157 (48.9%) from EMR clinics)  - Those patients aged 15-year old and above, nonilliterate, and Malaysian citizens   - 98 male   223 female | 14 public primary healthcare facilities (clinics) | - Patient satisfaction, (including communication) | - Higher/improved patient satisfaction and communication in clinics using EMR. |
| 18 | Krist et al. (2011) [42]  United States | - Develop and assess the adoption of an Interactive Preventive Health Record (IPHR) designed to engage patients in preventive care and health promotion. | Development of an IPHR, which generates tailored patient recommendations based on preventive care guidelines using electronic medical records (EMRs). | Randomized Controlled Trial & Usability Tests and Focus Groups  Mixed methods design (quantitative and qualitative) | Practice appointment and IPHR databases | Adult patients | 14 primary care practices involved in the Virginia Ambulatory Care Outcomes Network (ACORN) | - IPHR usage rate - Integration of IPHR into practice workflow for various purposes, including visit preparation, health behavior counseling, explaining test results, issuing reminders, prompting clinicians, and formulating personalized prevention plans. | - The IPHR successfully engaged patients in preventive care, with a usage rate ranging from 1.5% to 28.3% across practices. - Practices incorporated IPHR into workflow, utilizing it for multiple purposes related to patient care. - Technical advances: The IPHR model outlines five necessary components, including collecting patient information, integrating existing clinical data, interpreting patient information, providing personalized recommendations, and facilitating patient and clinician action. - Information resources: IPHR makes an open database connection (ODBC) to the EMR of the patient's personal clinician to extract relevant clinical data. Patients are shown their history, medications, immunizations, test dates, and results related to preventive care, allowing them to review, correct, and update their information. - The IPHR administers a brief health risk assessment to gather information not well recorded electronically, such as health behaviors and psychosocial measures. - The IPHR is designed as a longitudinal record and reminder system for patients and clinicians. It automatically re-queries the EMR to assess if patients are overdue for services, updates patient records, and generates email reminders and clinician summaries. The IPHR evolves with the patient's record, providing a sophisticated, longitudinal, personalized prevention plan. - A patient-centered personal health record provided individualized guidance and be adopted by busy primary care practices. |

**Reference list for the included studies cited within Multimedia Appendix 3:**

25- Zarcadoolas C, Vaughon WL, Czaja SJ, Levy J, Rockoff ML. Consumers' perceptions of patient-accessible electronic medical records. J Med Internet Res. Aug 26, 2013;15(8):e168. [doi: 10.2196/jmir.2507] [Medline: 23978618].

26- Wang L, He L, Tao Y, Sun L, Zheng H, Zheng Y, et al. Evaluating a web-based coaching program using electronic health records for patients with chronic obstructive pulmonary disease in China: randomized controlled trial. J Med Internet Res. Jul 21, 2017;19(7):e264. [doi: 10.2196/jmir.6743] [Medline: 28733270].

27- Nazi KM, Hogan TP, McInnes DK, Woods SS, Graham G. Evaluating patient access to Electronic Health Records: results from a survey of veterans. Med Care. Mar 2013;51(3 Suppl 1):S52-S56. [doi: 10.1097/MLR.0b013e31827808db] [Medline: 23407012].

28- Suija K, Mardo LA, Laidoja R, Nahkur S, Parvelo A, Kalda R. Experiences and expectation with the use of health data: a qualitative interview study in primary care. BMC Prim Care. Jun 23, 2022;23(1):159. [FREE Full text] [doi: 10.1186/s12875-022-01764-1] [Medline: 35739479].

29- Wass S, Vimarlund V, Ros A. Exploring patients' perceptions of accessing electronic health records: innovation in healthcare. Health Informatics J. Mar 2019;25(1):203-215. [doi: 10.1177/1460458217704258] [Medline: 28457195].

30- Spratt SE, Ravneberg D, Derstine B, Granger BB. Feasibility of electronic health record integration of a SMART application to facilitate patient-provider communication for medication management. Comput Inform Nurs. Aug 01, 2022;40(8):538-546. [doi: 10.1097/CIN.0000000000000891] [Medline: 35234708].

31- van der Vaart R, Drossaert CH, Taal E, Drossaers-Bakker KW, Vonkeman HE, van de Laar MA. Impact of patient-accessible electronic medical records in rheumatology: use, satisfaction and effects on empowerment among patients. BMC Musculoskelet Disord. Mar 26, 2014;15:102. [FREE Full text] [doi: 10.1186/1471-2474-15-102] [Medline: 24673997].

32- Klein TM, Augustin M, Kirsten N, Otten M. Attitudes towards using electronic health records of patients with psoriasis and dermatologists: a cross-sectional study. BMC Med Inform Decis Mak. Dec 30, 2020;20(1):344. [doi: 10.1186/s12911-020-01302-y] [Medline: 33380329].

33- Ryu B, Kim N, Heo E, Yoo S, Lee K, Hwang H, et al. Impact of an electronic health record-integrated personal health record on patient participation in health care: development and randomized controlled trial of MyHealthKeeper. J Med Internet Res. Dec 07, 2017;19(12):e401. [FREE Full text] [doi: 10.2196/jmir.8867] [Medline: 29217503].

34- Haggstrom DA, Saleem JJ, Russ AL, Jones J, Russell SA, Chumbler NR. Lessons learned from usability testing of the VA's personal health record. J Am Med Inform Assoc. Dec 2011;18 Suppl 1(Suppl 1):i13-i17. [FREE Full text] [doi: 10.1136/amiajnl-2010-000082] [Medline: 21984604].

35- Hanna L, Gill SD, Newstead L, Hawkins M, Osborne RH. Patient perspectives on a personally controlled electronic health record used in regional Australia. Health Inf Manag. Jan 2017;46(1):42-48. [doi: 10.1177/1833358316661063] [Medline: 27486184].

36- Fuller TE, Pong DD, Piniella N, Pardo M, Bessa N, Yoon C, et al. Interactive digital health tools to engage patients and caregivers in discharge preparation: implementation study. J Med Internet Res. Apr 28, 2020;22(4):e15573. [FREE Full text] [doi: 10.2196/15573] [Medline: 32343248].

37- Moll J, Rexhepi H, Cajander Å, Grünloh C, Huvila I, Hägglund M, et al. Patients' experiences of accessing their electronic health records: national patient survey in Sweden. J Med Internet Res. Nov 01, 2018;20(11):e278. [FREE Full text] [doi: 10.2196/jmir.9492] [Medline: 30389647].

38- Wolff JL, Darer JD, Berger A, Clarke D, Green JA, Stametz RA, et al. Inviting patients and care partners to read doctors' notes: OpenNotes and shared access to electronic medical records. J Am Med Inform Assoc. Apr 01, 2017;24(e1):e166-e172. [FREE Full text] [doi: 10.1093/jamia/ocw108] [Medline: 27497795].

39- Mák G, Smith Fowler H, Leaver C, Hagens S, Zelmer J. The effects of web-based patient access to laboratory results in British Columbia: a patient survey on comprehension and anxiety. J Med Internet Res. Aug 04, 2015;17(8):e191. [doi: 10.2196/jmir.4350] [Medline: 26242801].

40- Wagner PJ, Dias J, Howard S, Kintziger KW, Hudson MF, Seol YH, et al. Personal health records and hypertension control: a randomized trial. J Am Med Inform Assoc. 2012;19(4):626-634. [doi: 10.1136/amiajnl-2011-000349] [Medline: 22234404].

41- Ibrahim AA, Ahmad Zamzuri MI, Ismail R, Ariffin AH, Ismail A, Muhamad Hasani MH, et al. The role of electronic medical records in improving health care quality: a quasi-experimental study. Medicine (Baltimore). Jul 29, 2022;101(30):e29627. [doi: 10.1097/MD.0000000000029627] [Medline: 35905245].

42- Krist AH, Peele E, Woolf SH, Rothemich SF, Loomis JF, Longo DR, et al. Designing a patient-centered personal health record to promote preventive care. BMC Med Inform Decis Mak. Nov 24, 2011;11:73. [doi: 10.1186/1472-6947-11-73] [Medline: 22115059].
