# Supplementary material for: The Impact of Patient Access to Electronic Health Records on Health Care Engagement: Systematic Review
Source: J Med Internet Res. 2024 Nov 20;26:e56473. doi: 10.2196/56473 (PMC11618012; doi:10.2196/56473)
Supplement: Multimedia Appendix 4 [file jmir_v26i1e56473_app4.doc]

***Multimedia Appendix 4: Quality assessment of the included studies using the MMAT (2018)^a^**

| **First Author, Year** | 1. **^b^ Qualitative** | | | | | **2.,3.,4. Quantitative** | | | | | | | | | | | | | | | **5. Mixed Methods** | | | | | **Quality of the study** |
| --- | --- | --- | --- | --- | --- | --- | --- | --- | --- | --- | --- | --- | --- | --- | --- | --- | --- | --- | --- | --- | --- | --- | --- | --- | --- | --- |
|  |  |  |  |  |  | 1. **Randomized controlled trials (RCTs)** | | | | | 1. **Non-randomized studies** | | | | | 1. **Descriptive studies** | | | | |  |  |  |  |  |  |
| **References in Systematic review**  Fuller, 2020 [36]  Haggstrom, 2011 [34]  Hanna, 2017 [35]  Ibrahim , 2022 [41]  Klein, 2020 [32]  Krist, 2011 [42]  Mák, 2015 [39]  Moll, 2018 [37]  Nazi, 2013 [27]  Ryu, 2017 [33]  Spratt (2022) [30]  Suija (2022) [28]  van Der Vaart, 2014 [31]  Wagner, 2012 [40]  Wang, 2017 [26]  Wass, 2019 [29]  Wolff, 2017 [38]  Zarcadoolas, 2013 [25] | **1.1^c^**  Yes  No  Yes | **1.2**  Yes  Yes  Yes | **1.3**  Yes  No  Yes | **1.4**  Yes  No  No | **1.5**  Yes  Yes  Yes | **2.1**  Yes  Yes  Yes | **2.2**  Yes  Yes  Yes | **2.3**  Yes  Yes  Yes | **2.4**  Yes  No  Yes | **2.5**  Yes  Yes  Yes | **3.1**  Yes  Yes  Yes | **3.2**  Yes  Yes  Yes | **3.3**  Yes  Yes  Yes | **3.4**  Yes  No  Yes | **3.5**  Yes  Yes  Yes | **4.1**  Yes  Yes  Yes  Yes | **4.2**  Yes  Yes  Yes  Yes | **4.3**  Yes  Yes  Yes  Yes | **4.4**  Yes  Yes  Yes  Yes | **4.5**  Yes  Yes  Yes  Yes | **5.1**  Yes  Yes  Yes  Yes  Yes | **5.2**  Yes  Yes  Yes  Yes  Yes | **5.3**  Yes  Yes  Yes  Yes  Yes | **5.4**  Yes  Yes  Yes  Yes  Yes | **5.5**  Yes  Yes  Yes  Yes  Yes | High  High  High  High  High  High  Medium  High  High  High  High  Low  High  Medium  High  High  High  Medium |

^a^MMAT: Mixed Methods Appraisal Tool

^b^1., 2., 3., 4., 5.: Sections of the MMAT to evaluate studies.

^c^1.1-5.5: items in each of the MMAT sections used to evaluate the qualitative quantitative, and mixed methods studies.

*Note: all previous studies answered ‘Yes’ to the questions that related to the screening of the MMAT:

S.1. Are there clear research questions?

S.2. Do the collected data allow to address the research questions?

**Reference list for the included studies cited within Multimedia Appendix 4:**

25- Zarcadoolas C, Vaughon WL, Czaja SJ, Levy J, Rockoff ML. Consumers' perceptions of patient-accessible electronic medical records. J Med Internet Res. Aug 26, 2013;15(8):e168. [doi: 10.2196/jmir.2507] [Medline: 23978618].

26- Wang L, He L, Tao Y, Sun L, Zheng H, Zheng Y, et al. Evaluating a web-based coaching program using electronic health records for patients with chronic obstructive pulmonary disease in China: randomized controlled trial. J Med Internet Res. Jul 21, 2017;19(7):e264. [doi: 10.2196/jmir.6743] [Medline: 28733270].

27- Nazi KM, Hogan TP, McInnes DK, Woods SS, Graham G. Evaluating patient access to Electronic Health Records: results from a survey of veterans. Med Care. Mar 2013;51(3 Suppl 1):S52-S56. [doi: 10.1097/MLR.0b013e31827808db] [Medline: 23407012].

28- Suija K, Mardo LA, Laidoja R, Nahkur S, Parvelo A, Kalda R. Experiences and expectation with the use of health data: a qualitative interview study in primary care. BMC Prim Care. Jun 23, 2022;23(1):159. [FREE Full text] [doi: 10.1186/s12875-022-01764-1] [Medline: 35739479].

29- Wass S, Vimarlund V, Ros A. Exploring patients' perceptions of accessing electronic health records: innovation in healthcare. Health Informatics J. Mar 2019;25(1):203-215. [doi: 10.1177/1460458217704258] [Medline: 28457195].

30- Spratt SE, Ravneberg D, Derstine B, Granger BB. Feasibility of electronic health record integration of a SMART application to facilitate patient-provider communication for medication management. Comput Inform Nurs. Aug 01, 2022;40(8):538-546. [doi: 10.1097/CIN.0000000000000891] [Medline: 35234708].

31- van der Vaart R, Drossaert CH, Taal E, Drossaers-Bakker KW, Vonkeman HE, van de Laar MA. Impact of patient-accessible electronic medical records in rheumatology: use, satisfaction and effects on empowerment among patients. BMC Musculoskelet Disord. Mar 26, 2014;15:102. [FREE Full text] [doi: 10.1186/1471-2474-15-102] [Medline: 24673997].

32- Klein TM, Augustin M, Kirsten N, Otten M. Attitudes towards using electronic health records of patients with psoriasis and dermatologists: a cross-sectional study. BMC Med Inform Decis Mak. Dec 30, 2020;20(1):344. [doi: 10.1186/s12911-020-01302-y] [Medline: 33380329].

33- Ryu B, Kim N, Heo E, Yoo S, Lee K, Hwang H, et al. Impact of an electronic health record-integrated personal health record on patient participation in health care: development and randomized controlled trial of MyHealthKeeper. J Med Internet Res. Dec 07, 2017;19(12):e401. [FREE Full text] [doi: 10.2196/jmir.8867] [Medline: 29217503].

34- Haggstrom DA, Saleem JJ, Russ AL, Jones J, Russell SA, Chumbler NR. Lessons learned from usability testing of the VA's personal health record. J Am Med Inform Assoc. Dec 2011;18 Suppl 1(Suppl 1):i13-i17. [FREE Full text] [doi: 10.1136/amiajnl-2010-000082] [Medline: 21984604].

35- Hanna L, Gill SD, Newstead L, Hawkins M, Osborne RH. Patient perspectives on a personally controlled electronic health record used in regional Australia. Health Inf Manag. Jan 2017;46(1):42-48. [doi: 10.1177/1833358316661063] [Medline: 27486184].

36- Fuller TE, Pong DD, Piniella N, Pardo M, Bessa N, Yoon C, et al. Interactive digital health tools to engage patients and caregivers in discharge preparation: implementation study. J Med Internet Res. Apr 28, 2020;22(4):e15573. [FREE Full text] [doi: 10.2196/15573] [Medline: 32343248].

37- Moll J, Rexhepi H, Cajander Å, Grünloh C, Huvila I, Hägglund M, et al. Patients' experiences of accessing their electronic health records: national patient survey in Sweden. J Med Internet Res. Nov 01, 2018;20(11):e278. [FREE Full text] [doi: 10.2196/jmir.9492] [Medline: 30389647].

38- Wolff JL, Darer JD, Berger A, Clarke D, Green JA, Stametz RA, et al. Inviting patients and care partners to read doctors' notes: OpenNotes and shared access to electronic medical records. J Am Med Inform Assoc. Apr 01, 2017;24(e1):e166-e172. [FREE Full text] [doi: 10.1093/jamia/ocw108] [Medline: 27497795].

39- Mák G, Smith Fowler H, Leaver C, Hagens S, Zelmer J. The effects of web-based patient access to laboratory results in British Columbia: a patient survey on comprehension and anxiety. J Med Internet Res. Aug 04, 2015;17(8):e191. [doi: 10.2196/jmir.4350] [Medline: 26242801].

40- Wagner PJ, Dias J, Howard S, Kintziger KW, Hudson MF, Seol YH, et al. Personal health records and hypertension control: a randomized trial. J Am Med Inform Assoc. 2012;19(4):626-634. [doi: 10.1136/amiajnl-2011-000349] [Medline: 22234404].

41- Ibrahim AA, Ahmad Zamzuri MI, Ismail R, Ariffin AH, Ismail A, Muhamad Hasani MH, et al. The role of electronic medical records in improving health care quality: a quasi-experimental study. Medicine (Baltimore). Jul 29, 2022;101(30):e29627. [doi: 10.1097/MD.0000000000029627] [Medline: 35905245].

42- Krist AH, Peele E, Woolf SH, Rothemich SF, Loomis JF, Longo DR, et al. Designing a patient-centered personal health record to promote preventive care. BMC Med Inform Decis Mak. Nov 24, 2011;11:73. [doi: 10.1186/1472-6947-11-73] [Medline: 22115059].
